# Supplementary material for: CNS-wide repopulation by hematopoietic-derived microglia-like cells corrects progranulin deficiency in mice
Source: Nat Commun. 2024 Jul 5;15:5654. doi: 10.1038/s41467-024-49908-4 (PMC11226701; doi:10.1038/s41467-024-49908-4)
Supplement: Supplementary file 1 — Supplementary Information [file 41467_2024_49908_MOESM1_ESM.pdf]

CNS-wide repopulation by hematopoietic-derived microglia-like cells corrects progranulin deficiency in mice

Author list

Pasqualina Colella[1*], Ruhi Sayana[1], Maria Valentina Suarez-Nieto[1], Jolanda Sarno[2,3], Kwamina Nyame[4,5,6], Jian Xiong[4,5,6], Luisa Natalia Pimentel Vera[1], Jessica Arozqueta Basurto[1], Marco Corbo[7], Anay Limaye1[1,7], Kara L. Davis[2], Monther Abu-Remaileh[4,5,6] Natalia Gomez-Ospina[1*].

Affiliations

[1]Department of Pediatrics, Stanford University School of Medicine, Stanford, CA, 94305, USA.
[2]Hematology, Oncology, Stem Cell Transplant, and Regenerative Medicine, Department of Pediatrics, Stanford University, Stanford, CA, 94305, USA.
[3]Tettamanti Center, Fondazione IRCCS San Gerardo dei Tintori, 20900, Monza, Italy.
[4]Department of Chemical Engineering, Stanford University, Stanford, CA ,94305, USA.
[5]Department of Genetics, Stanford University, Stanford, CA, 94305, USA.
[6]The Institute for Chemistry, Engineering and Medicine for Human Health (Sarafan ChEM-H), Stanford University, Stanford, CA, 94305, USA.
[7]MedGenome, Inc, 348 Hatch Dr, Foster City, CA, 94404, USA.

*Corresponding authors:

Pasqualina Colella, PhD pcolella@stanford.edu

Natalia Gomez-Ospina MD, PhD: gomezosp@stanford.edu

Table of contents

Supplementary Figure 1 1
Supplementary Figure 2 3
Supplementary Figure 3 4
Supplementary Figure 4 5
Supplementary Figure 5 6
Supplementary Figure 6 7
Supplementary Figure 7 9
Supplementary Figure 8 10
Supplementary Figure 9 11
Supplementary Figure 10 13
Supplementary Figure 11 15
Supplementary Figure 12 16
Supplementary Figure 13 18
Supplementary Figure 14 19
Supplementary Figure 15 20
Supplementary Figure 16 21
Supplementary Figure 17 22

Supplementary Table 1 23
Supplementary Table 2 24
Supplementary Table 3 25

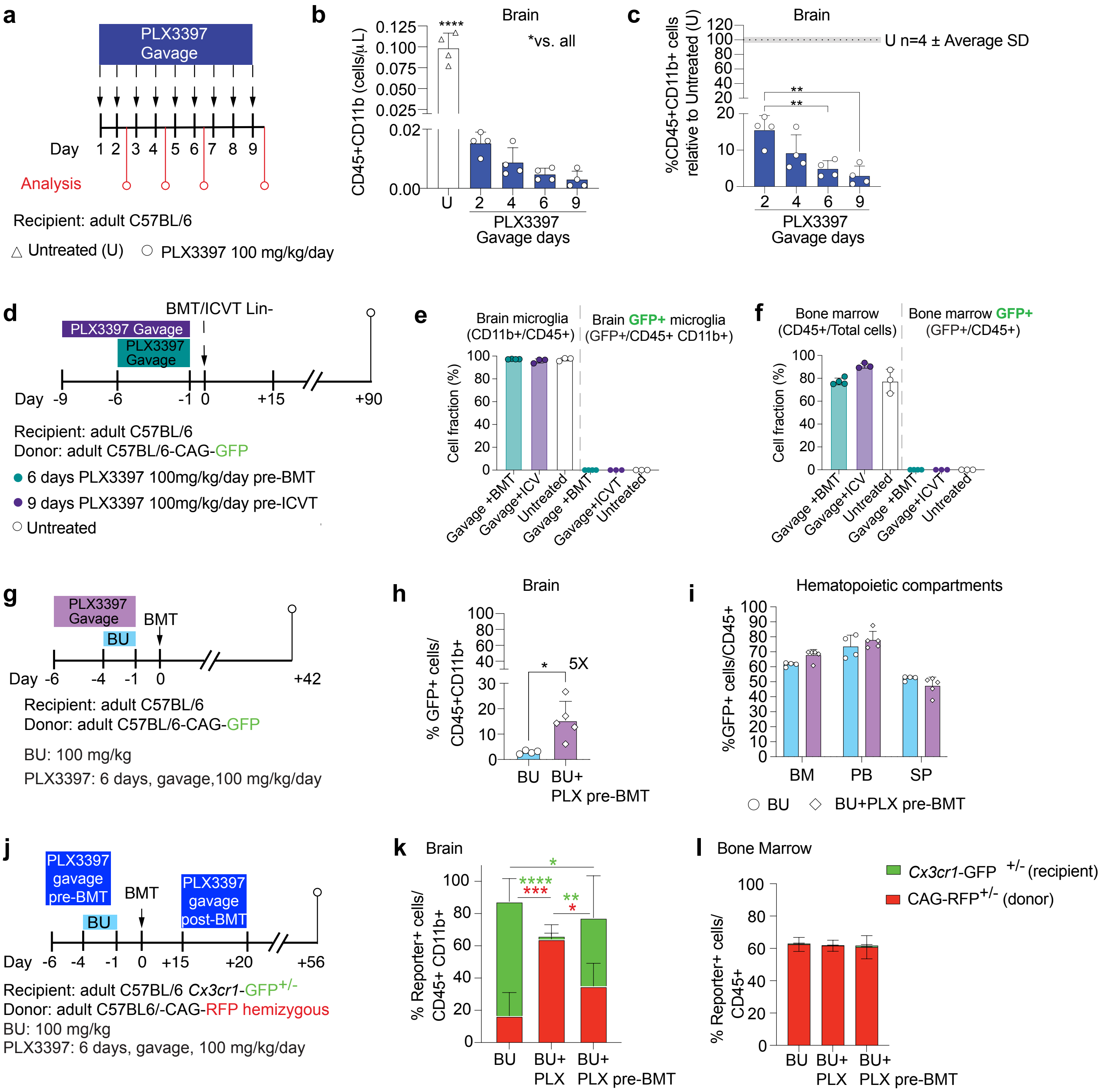
a
PLX3397 Gavage
Day 1 2 3 4 5 6 7 8 9
Analysis
Recipient: adult C57BL/6
△ Untreated (U) ○ PLX3397 100 mg/kg/day
b
Brain
*vs. all
****
CD45+CD11b (cells/μL)
U 2 4 6 9
PLX3397 Gavage days
c
Brain
U n=4 ± Average SD
%CD45+CD11b+ cells relative to Untreated (U)
**
**
2 4 6 9
PLX3397 Gavage days
d
BMT/ICVT Lin-
PLX3397 Gavage
PLX3397 Gavage
Day -9 -6 -1 0 +15 +90
Recipient: adult C57BL/6
Donor: adult C57BL/6-CAG-GFP
6 days PLX3397 100mg/kg/day pre-BMT
9 days PLX3397 100mg/kg/day pre-ICVT
Untreated
e
Brain microglia (CD11b+/CD45+)
Brain GFP+ microglia (GFP+/CD45+ CD11b+)
Cell fraction (%)
Gavage +BMT
Gavage+ICV
Untreated
Gavage +BMT
Gavage+ICVT
Untreated
f
Bone marrow (CD45+/Total cells)
Bone marrow GFP+ (GFP+/CD45+)
Cell fraction (%)
Gavage +BMT
Gavage+ICV
Untreated
Gavage +BMT
Gavage+ICVT
Untreated
g
PLX3397 Gavage
BU
BMT
Day -6 -4 -1 0 +42
Recipient: adult C57BL/6
Donor: adult C57BL/6-CAG-GFP
BU: 100 mg/kg
PLX3397: 6 days, gavage,100 mg/kg/day
h
Brain
% GFP+ cells/ CD45+CD11b+
*
5X
BU
BU+ PLX pre-BMT
i
Hematopoietic compartments
%GFP+ cells/CD45+
BM PB SP
○ BU ◇ BU+PLX pre-BMT
j
PLX3397 gavage pre-BMT
BU
BMT
PLX3397 gavage post-BMT
Day -6 -4 -1 0 +15 +20 +56
Recipient: adult C57BL/6 Cx3cr1-GFP+/-
Donor: adult C57BL6/-CAG-RFP hemizygous
BU: 100 mg/kg
PLX3397: 6 days, gavage, 100 mg/kg/day
k
Brain
*
****
***
**
*
% Reporter+ cells/ CD45+ CD11b+
BU
BU+ PLX
BU+ PLX pre-BMT
l
Bone Marrow
Cx3cr1-GFP +/- (recipient)
CAG-RFP+/- (donor)
% Reporter+ cells/ CD45+
BU
BU+ PLX
BU+ PLX pre-BMT

Supplementary Figure 1. Optimization of a PLX3397 conditioning regimen for microglia depletion and replacement.
a-c Analyses of microglia depletion in the brain of adult C57BL/6 mice conditioned with PLX3397 (PLX) by oral gavage for 2, 4, 6, or 9 consecutive days at 100 mg/kg/day (n = 4 mice/cohort). Untreated age- and sex-matched mice were used as control (U, n = 4 mice/cohort). **a** Experimental timeline showing the PLX dosing and time points of analysis. **b-c** Absolute count of CD45+CD11b+ cells in the brain measured by flow cytometry and expressed as either absolute values (**b**), or percentages relative to Untreated mice (**c**, U = 100%, grey line). *p-value vs Untreated mice (U). **d-i** Analysis of donor cell engraftment in brain and hematopoietic compartments of adult C57BL/6 mice treated with the optimized PLX regimen and receiving either intravenous bone marrow transplant (BMT) or intracerebroventricular transplant (ICVT) of Lineage negative (Lin-) bone marrow cells, isolated from homozygous C57BL/6-CAG-GFP mice. **d** Experimental timeline. Untreated: age- and sex-matched control mice. PLX was administered by oral gavage (100 mg/kg/day for 6 or 9 days). **e-f** Analysis of donor chimerism in measured by flow cytometry in the brain (**e**) and bone marrow (**f**) at study end. The left panels show the relative frequency of CD11b+/ CD45+ cells in the brain (**e**) and CD45+ hematopoietic cells in the bone marrow (**f**) at the same time point. Mouse numbers (n): BMT n = 4, ICV Lin- n = 3, Untreated n = 3. **g** Experimental timeline showing the myeloablation of C57BL/6 mice with busulfan (BU, 100 mg/kg) and 6-day PLX conditioning (600 mg/kg, 100 mg/kg/day) before BMT (pre-BMT). Follow-up was 1.5 months after BMT. **h-i** Fraction of transplant-derived GFP+ CD45+CD11b+ cells measured by flow cytometry at study end in brain (**h**) and hematopoietic compartments [**i**, bone marrow (BM), peripheral blood (PB), and spleen (SP)]. BU n=4, BU + PLX pre-BMT n=5. **j-l** Analysis of donor chimerism in brain and bone marrow of adult *Cx3cr1*-GFP$^{+/-}$ mice conditioned with BU, or BU+ PLX. PLX was given either pre- or post-BMT. Whole bone marrow was isolated from adult C57BL/6-CAG-RFP hemizygous mice. **j** Experimental timeline. Follow-up was 2 months after BMT. **k-l** Fraction of transplant-derived RFP+ and recipient *Cx3cr1*-GFP$^{+/-}$ cells measured in the brain (**k**) and bone marrow (**l**) by flow cytometry. BU n = 4, BU + PLX n = 4, BU + PLX pre-BMT n = 3. **a-l** Data are reported as Mean ± SD. Source data are provided as a Source Data file. Statistical analysis: *p < 0.05, **p < 0.01, ***p < 0.001, ****p < 0.0001, the exact p-values of all comparisons are reported in the Source Data file; **b-c** One-way ANOVA with Tukey post-hoc; **h** Two-tailed unpaired t-test; **i, k-l** Two-way ANOVA with Tukey post-hoc. **k** The green asterisks show statistically significant differences in the fraction of recipient *Cx3cr1*-GFP+ cells between the indicated groups; the red asterisks show statistically significant differences in the fraction of BMT-derived RFP+ cells between the indicated groups.

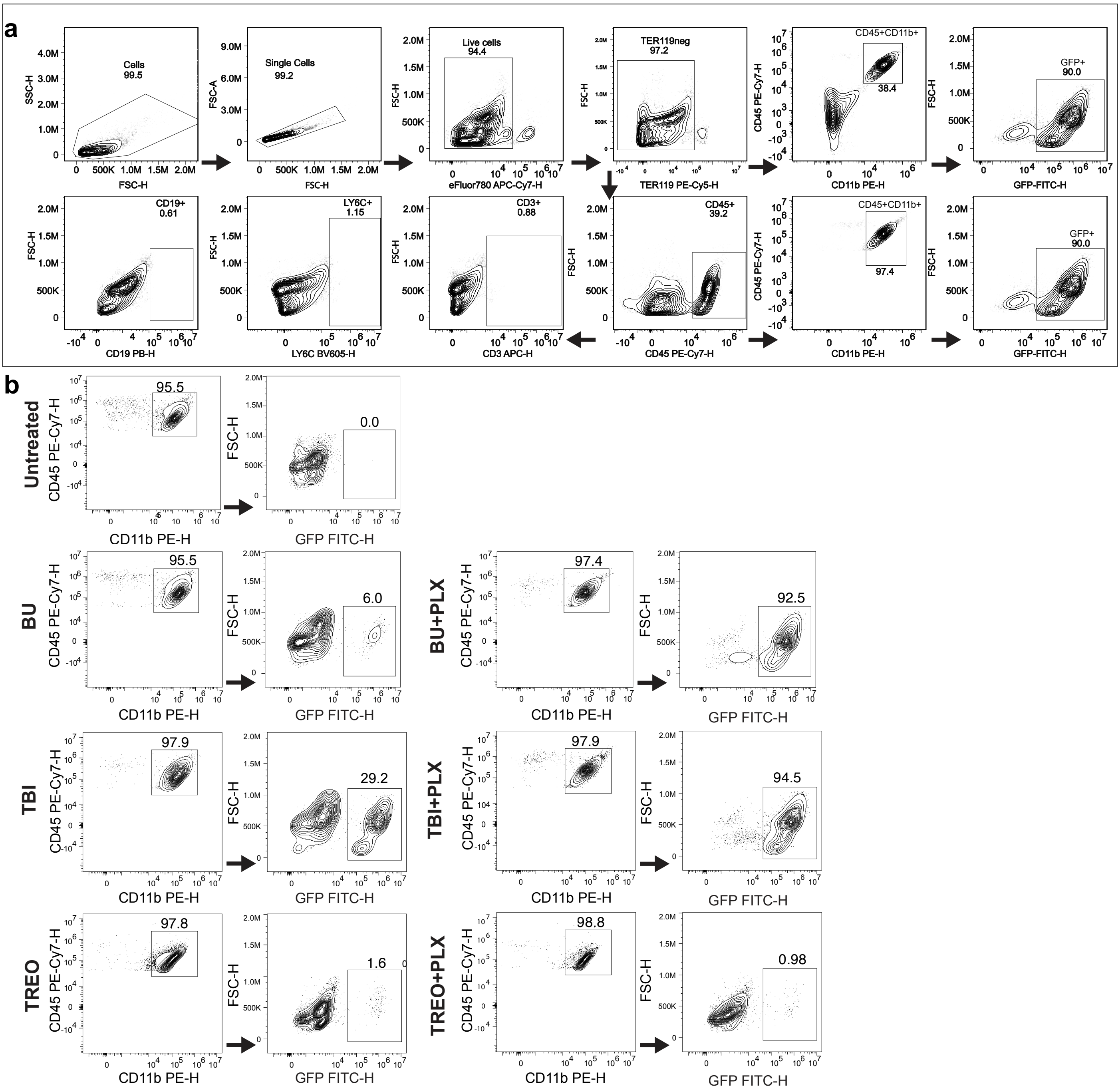

Supplementary Figure 2. Gating strategy and analysis of brain donor chimerism.
a Representative gating strategy of cells isolated from the brain and analyzed by flow cytometry. The representative flow plots depict the gating strategy to get the percentage of transplant-derived GFP+ cells measured in the brain of an adult C57BL/6 mouse transplanted with total bone marrow from C57BL/6-CAG-GFP mice and conditioned as depicted in **Fig. 1a.** The gating strategy to quantify pro-inflammatory hematopoietic cells infiltrated in the brain is also depicted (Ly6C+ cells, CD19+ B cells and CD3+ T cells, data depicted in **Fig. 1f**). The time point of the depicted analyses is 7 months after bone marrow transplant. **b** Representative flow plots relative to **Fig. 1 a-b.** The plots depict the percentage of transplant-derived GFP+ cells measured in the brain of adult C57BL/6 mice transplanted with total bone marrow from C57BL/6-CAG-GFP mice and conditioned with either total body irradiation (TBI) +/- PLX3397, busulfan (BU) +/- PLX3397 or treosulfan (TREO) +/- PLX3397, one mouse/cohort is depicted. The time point of the analyses is three months after bone marrow transplant.

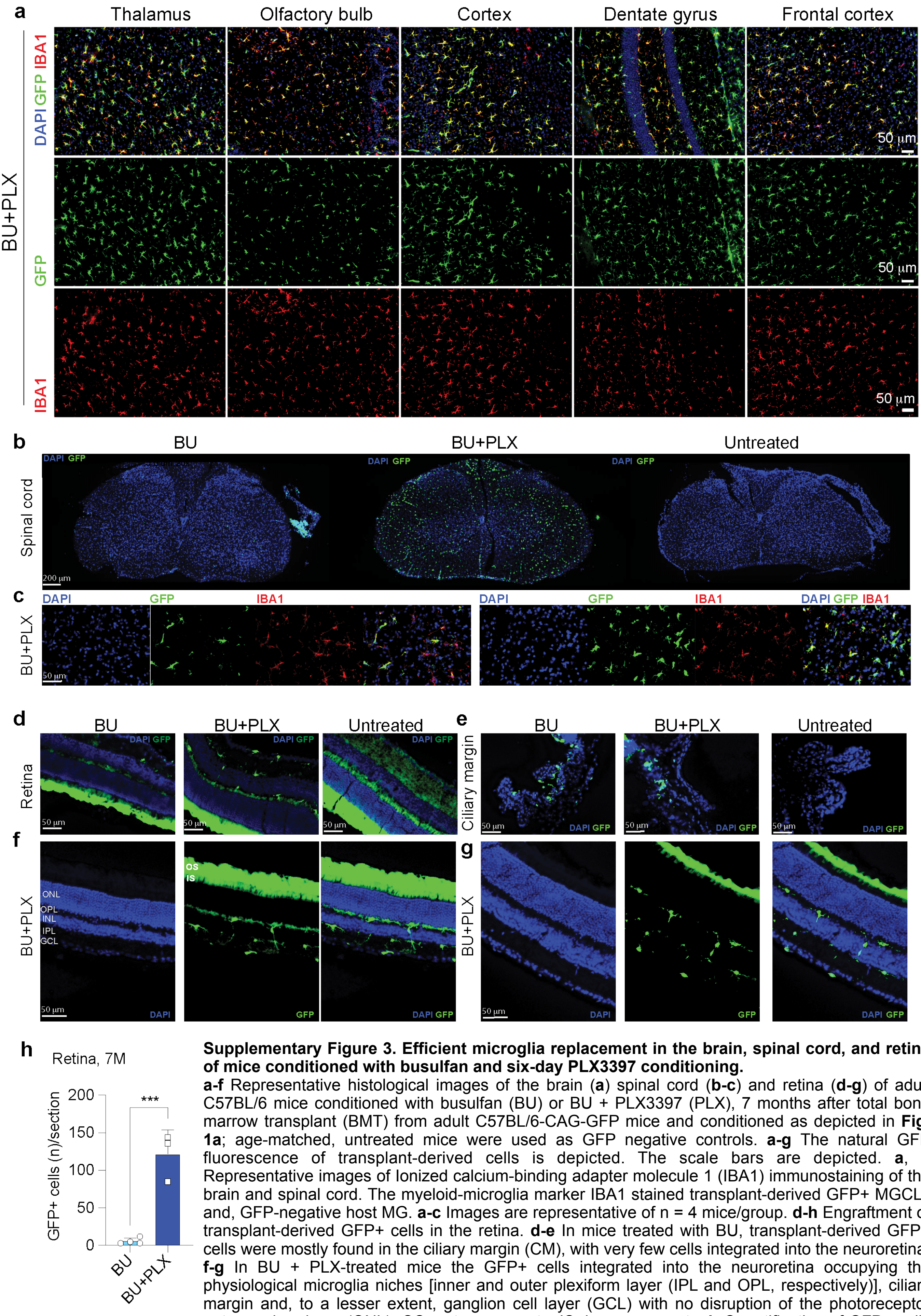

Supplementary Figure 3. Efficient microglia replacement in the brain, spinal cord, and retina of mice conditioned with busulfan and six-day PLX3397 conditioning.

a-f Representative histological images of the brain (**a**) spinal cord (**b-c**) and retina (**d-g**) of adult C57BL/6 mice conditioned with busulfan (BU) or BU + PLX3397 (PLX), 7 months after total bone marrow transplant (BMT) from adult C57BL/6-CAG-GFP mice and conditioned as depicted in **Fig. 1a**; age-matched, untreated mice were used as GFP negative controls. **a-g** The natural GFP fluorescence of transplant-derived cells is depicted. The scale bars are depicted. **a**, **c** Representative images of Ionized calcium-binding adapter molecule 1 (IBA1) immunostaining of the brain and spinal cord. The myeloid-microglia marker IBA1 stained transplant-derived GFP+ MGCLs and, GFP-negative host MG. **a-c** Images are representative of n = 4 mice/group. **d-h** Engraftment of transplant-derived GFP+ cells in the retina. **d-e** In mice treated with BU, transplant-derived GFP+ cells were mostly found in the ciliary margin (CM), with very few cells integrated into the neuroretina. **f-g** In BU + PLX-treated mice the GFP+ cells integrated into the neuroretina occupying the physiological microglia niches [inner and outer plexiform layer (IPL and OPL, respectively)], ciliary margin and, to a lesser extent, ganglion cell layer (GCL) with no disruption of the photoreceptor outer nuclear layer (ONL); OS: outer segments; IS: inner segments. **h** Quantification of GFP+ cells (n)/retinal section in mice treated with BU and BU + PLX. Representative images (d-g) and quantification (h) derive from BU n = 4 mice and BU + PLX n=3 mice. Data are reported as Mean ± SD. Source data are provided as a Source Data file. Statistical analysis: two-tailed unpaired t-test, p=0.0007.

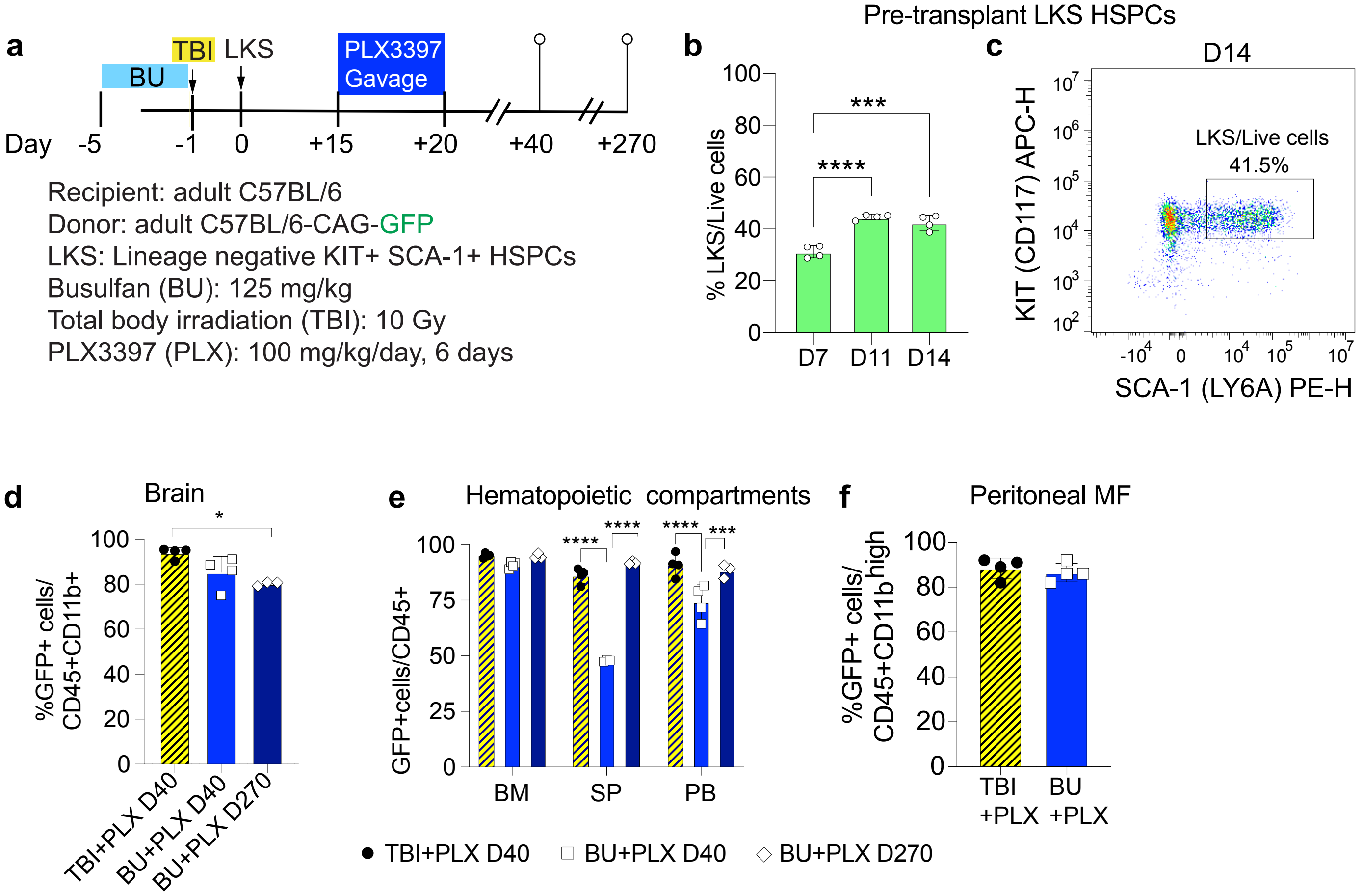

Supplementary Figure 4. Kinetics of HSPC engraftment using busulfan and six-day PLX3397 conditioning. **a** Experimental timeline showing the conditioning and transplant of adult C57BL/6 mice with Lineage negative KIT+ SCA-1+ (LKS) hematopoietic stem and progenitor cells (HSPCs) isolated from adult homozygous C57BL/6-CAG-GFP mice and expanded in culture for 14 days. Follow-up was 40 (D40) or 270 (D270) days after transplant. Myeloablation was performed using either total body irradiation (TBI, 10 Gy) or busulfan (BU, 125 mg/kg). **b** Percentage of HPSCs positive for the stemness markers KIT (CD117) and SCA-1 quantified by flow cytometry at culture day 7 (D7), day 11 (D11), and day 14 (D14); n = 4 mouse donors time point (4 biological replicates). **c** Representative flow plot of LKS HPSCs at culture day 14. **d-f** Percentage of transplant-derived GFP+ cells in the brain (**d**), hematopoietic compartments [**e**, bone marrow (BM), spleen (SP) and peripheral blood (PB)] and, peritoneum (**f**) at the indicated time points. **b, d-f** Mouse numbers (n): day 40 n = 4 (except spleen for BU + PLX n = 3 mice), day 270 n = 3. Data are Mean ± SD. Source data are provided as a Source Data file. Statistical analysis: *p < 0.05, ***p < 0.001, ****p < 0.0001, the exact p-values of all comparisons are reported in the Source Data file; **b** One-way ANOVA with Tukey post-hoc; **d** Kruskal-Wallis with Dunn's post-hoc; **e** Two-way ANOVA with Sidak post-hoc; **f** Two-tailed unpaired t-test. MF: macrophages.

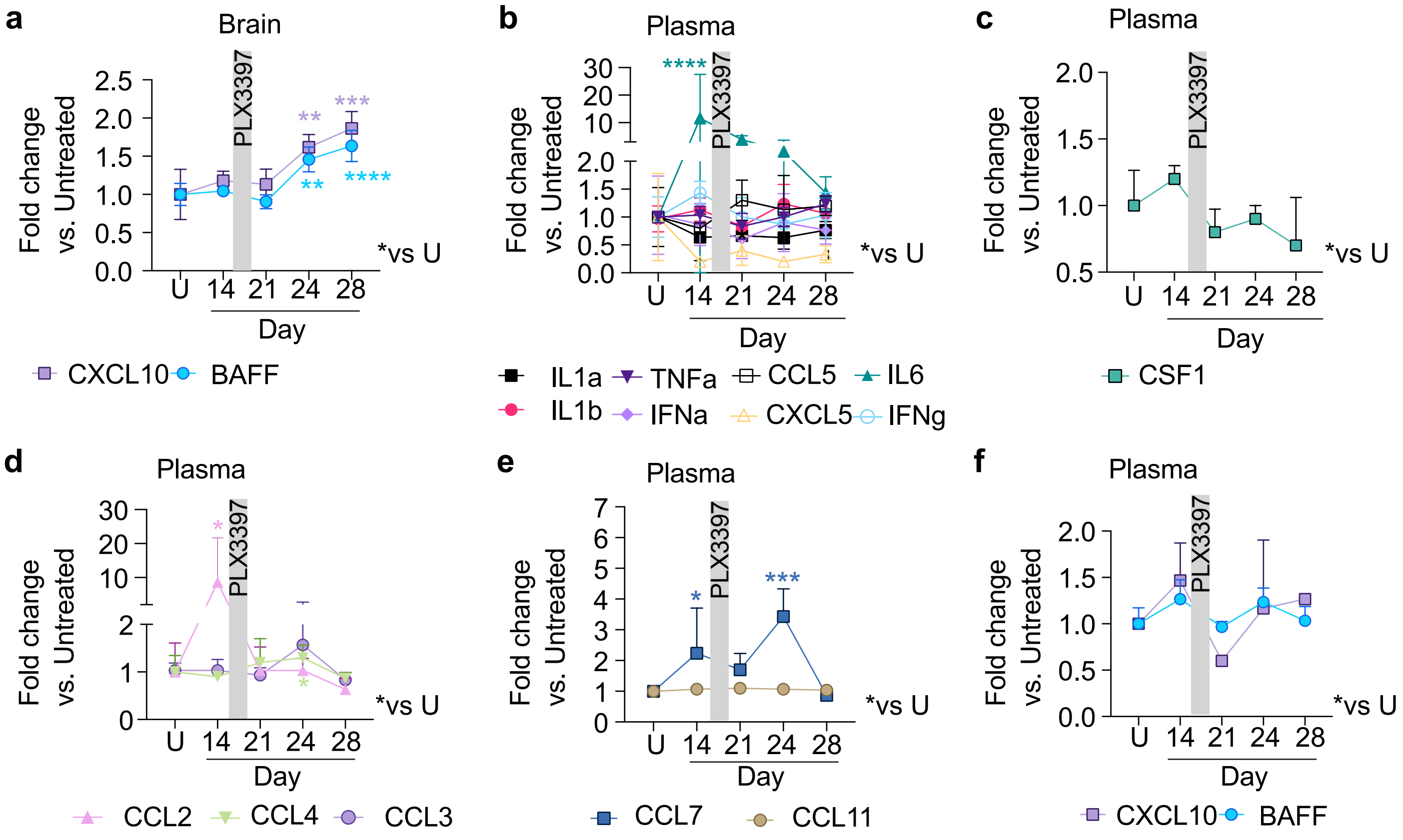

Supplementary Figure 5. Kinetics of cytokine production and donor cell engraftment using busulfan plus six-day PLX3397 conditioning.

a-f Cytokine analysis performed by 48-plex Luminex assay in brain (**a**) and plasma (**b-f**) of adult C57BL/6 mice conditioned with busulfan and PLX3397 and transplanted with total bone marrow from adult C57BL/6-CAG-GFP mice. The days indicate the time point after bone marrow transplant. The experimental scheme and timeline are depicted in **Fig. 2a**. Cytokine quantifications and statistics are relative to untreated mice (*p-value vs U). Grey bar: PLX3397 administration window. Mouse numbers (n): n = 3 per time point. **a-f** Data are Mean ± SD. Source data are provided as a Source Data file. Statistical analysis: *p < 0.05, **p < 0.01, ***p < 0.001, ****p < 0.0001, the exact p-values of all comparisons are reported in the Source Data file; **a-b, d-f** Two-way ANOVA vs Untreated with Dunnett post-hoc; **c** One-way ANOVA vs Untreated with Dunnett post-hoc.

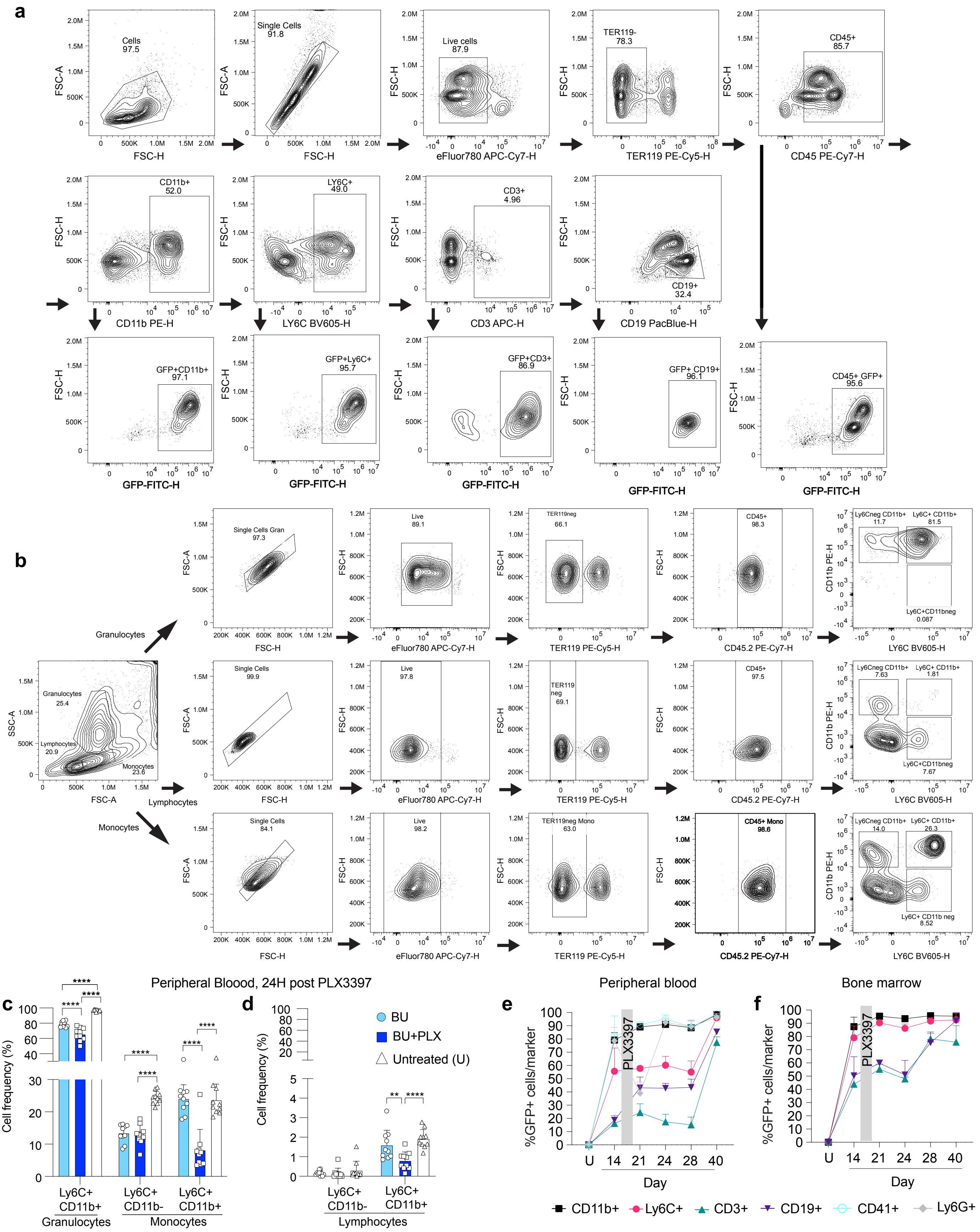

a
Cells 97.5
Single Cells 91.8
Live cells 87.9
TER119- 78.3
CD45+ 85.7
CD11b+ 52.0
LY6C+ 49.0
CD3+ 4.96
CD19+ 32.4
GFP+CD11b+ 97.1
GFP+Ly6C+ 95.7
GFP+CD3+ 86.9
GFP+ CD19+ 96.1
CD45+ GFP+ 95.6
FSC-A
FSC-H
eFluor780 APC-Cy7-H
TER119 PE-Cy5-H
CD45 PE-Cy7-H
CD11b PE-H
LY6C BV605-H
CD3 APC-H
CD19 PacBlue-H
GFP-FITC-H
b
Granulocytes 25.4
Lymphocytes 20.9
Monocytes 23.6
SSC-A
FSC-A
Granulocytes
Lymphocytes
Monocytes
Single Cells Gran 97.3
Live 89.1
TER119neg 66.1
CD45+ 98.3
Ly6Cneg CD11b+ 11.7
Ly6C+ CD11b+ 81.5
Ly6C+CD11bneg 0.087
Single Cells 99.9
Live 97.8
TER119 neg 69.1
CD45+ 97.5
Ly6Cneg CD11b+ 7.63
Ly6C+ CD11b+ 1.81
Ly6C+CD11bneg 7.67
Single Cells 84.1
Live 98.2
TER119neg Mono 63.0
CD45+ Mono 98.6
Ly6Cneg CD11b+ 14.0
Ly6C+ CD11b+ 26.3
Ly6C+ CD11b neg 8.52
CD45.2 PE-Cy7-H
CD11b PE-H
LY6C BV605-H
c
Peripheral Bloood, 24H post PLX3397
Cell frequency (%)
Ly6C+ CD11b+
Granulocytes
Ly6C+ CD11b-
Ly6C+ CD11b+
Monocytes
d
Cell frequency (%)
BU
BU+PLX
Untreated (U)
Ly6C+ CD11b-
Ly6C+ CD11b+
Lymphocytes
e
Peripheral blood
%GFP+ cells/marker
PLX3397
Day
U 14 21 24 28 40
f
Bone marrow
%GFP+ cells/marker
PLX3397
Day
U 14 21 24 28 40
CD11b+
Ly6C+
CD3+
CD19+
CD41+
Ly6G+

Supplementary Figure 6. Gating strategy and analysis of hematopoietic lineages and donor chimerism in bone marrow, spleen, and peripheral blood.

a Representative gating strategy of cells isolated from the hematopoietic compartments (bone marrow, spleen, and peripheral blood). The representative flow plots depict the gating strategy used to calculate the percentage of marker-positive cells out of CD45+ cells and, the percentage of transplant-derived GFP+ cells. The bone marrow from one adult C57BL/6 mouse transplanted with total bone marrow from C57BL/6-CAG-GFP mice and conditioned with busulfan + PLX3397 is depicted (BU+PLX, **Fig. 1a**). The time point of the analyses is 7 months after bone marrow transplant. **b-d** Analysis of Ly6C+ cells within lymphocyte, monocyte, and granulocyte lineages in the peripheral blood of adult C57BL/6 mice conditioned with busulfan (BU) or BU+PLX3397 (PLX) and transplanted with total bone marrow from C57BL/6-CAG-GFP mice (timeline shown in **Fig. 1a)**. The representative gating strategy distinguishes lymphocytes, monocytes, and granulocytes based on their relative size. The bar plots show the frequency of Ly6C+ lineages in the peripheral blood 21 days after bone marrow transplant (equivalent to 24 hours after PLX3397 withdrawal), with untreated mice used as controls. Data are reported as Mean ± SD; n = 10 mice/cohort. Source data are provided as a Source Data file. **e-f** Kinetics of hematopoietic lineage reconstitution in peripheral blood (**e**) and bone marrow (**f**) of adult C57BL/6 mice before and after BU + PLX conditioning and up to 40 days after transplant. The fraction of GFP+ myeloid cells (CD11b+, Ly6C+), platelets (CD41+), granulocytes (Ly6G+), T cells (CD3+), and B cells (CD19+) out of total CD45+ cells measured by flow cytometry is reported; n= 3 mice/time point. Statistical analyses: $**p < 0.01$, $****p < 0.0001$, the exact p-values of all comparisons are reported in the Source Data file; **c-d** Two-way ANOVA with Tukey post-hoc.

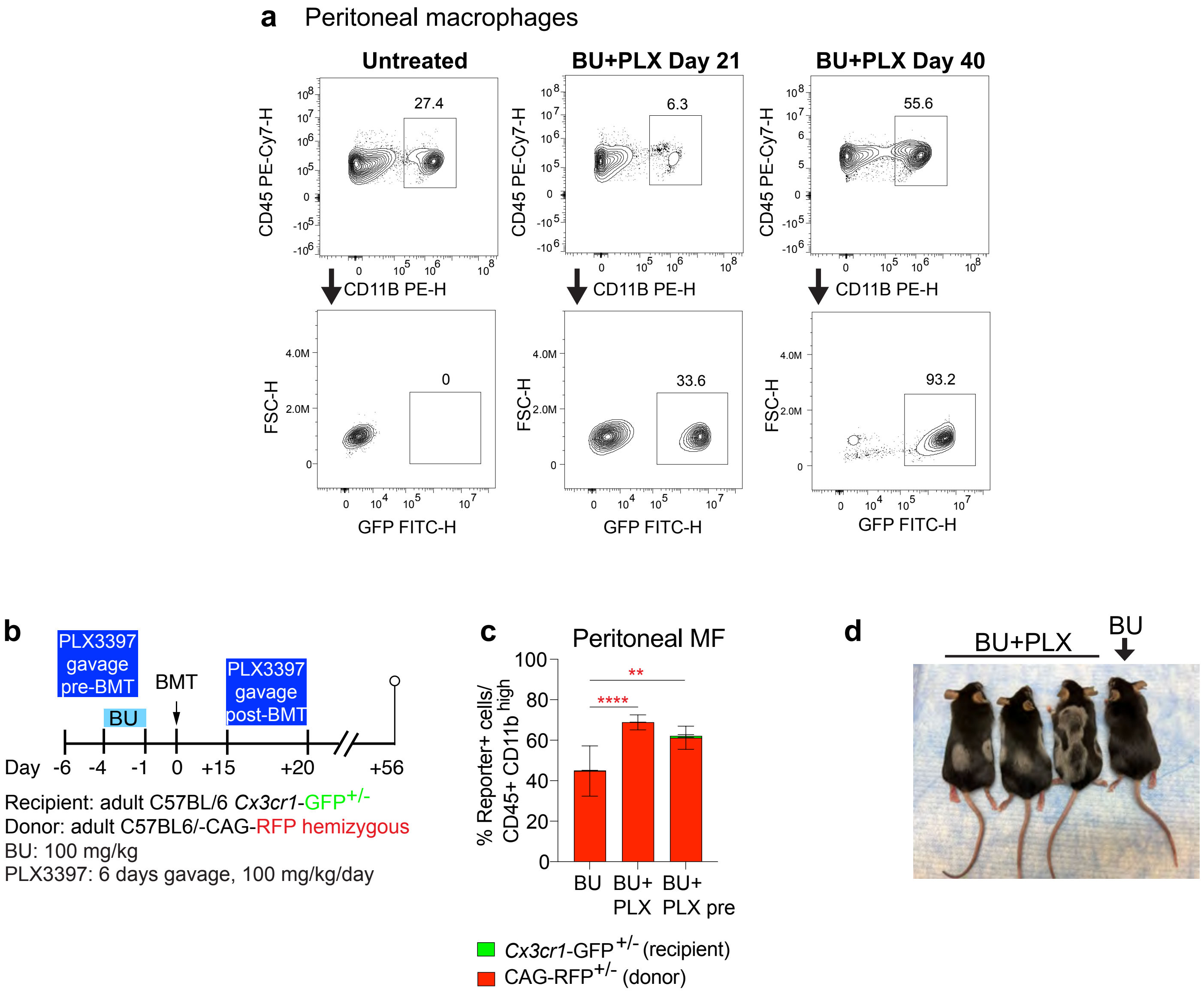

Supplementary Figure 7. Macrophage gating strategy and analysis of donor macrophage chimerism.
a Representative gating strategy of macrophages isolated from mouse organs and peritoneum. CD45+ CD11b+ cells are gated on total CD45+ cells as depicted in **Supp. Fig. 6a**. The flow plots depict the percentage of transplant-derived GFP+ CD45+ CD11b^{high} macrophages (MF) measured in the peritoneum of adult C57BL/6 mice conditioned with busulfan + PLX3397 (BU + PLX) and transplanted with total bone marrow from C57BL/6-CAG-GFP mice, one mouse/time point is depicted; the time points indicate the days after transplant. The representative flow plots are relative to the data depicted in **Fig. 3k. b-c** Analysis of CD45+ CD11b^{high} MF engraftment in the peritoneum of adult C57BL/6-*Cx3cr1*-GFP$^{+/-}$ mice treated with BU or BU+ PLX. PLX was given either before or after bone marrow transplant (BMT). **b** Experimental timeline. Follow-up was 2 months after BMT. **c** Fraction of transplant-derived RFP+ and recipient GFP+ MF measured by flow cytometry; mouse numbers (n): BU n = 4, BU + PLX n =4, BU + PLX pre-BMT n = 3. Data are Mean ± SD. Source data are provided as a Source Data file. Statistical analysis: **p < 0.01, ***p < 0.001; **c** Two-way ANOVA with Tukey post-hoc; the red asterisks show statistically significant differences in the fraction of BMT-derived RFP+ cells between the indicated groups. The exact p-values of all comparisons are reported in the Source Data file. **d** Representative image showing the patches of hair discoloration observed in adult C57BL/6 mice conditioned with BU + PLX but not the ones conditioned with BU only. The time point of the analyses is 7 months after transplant. The picture is representative of n=10 mice/cohort.

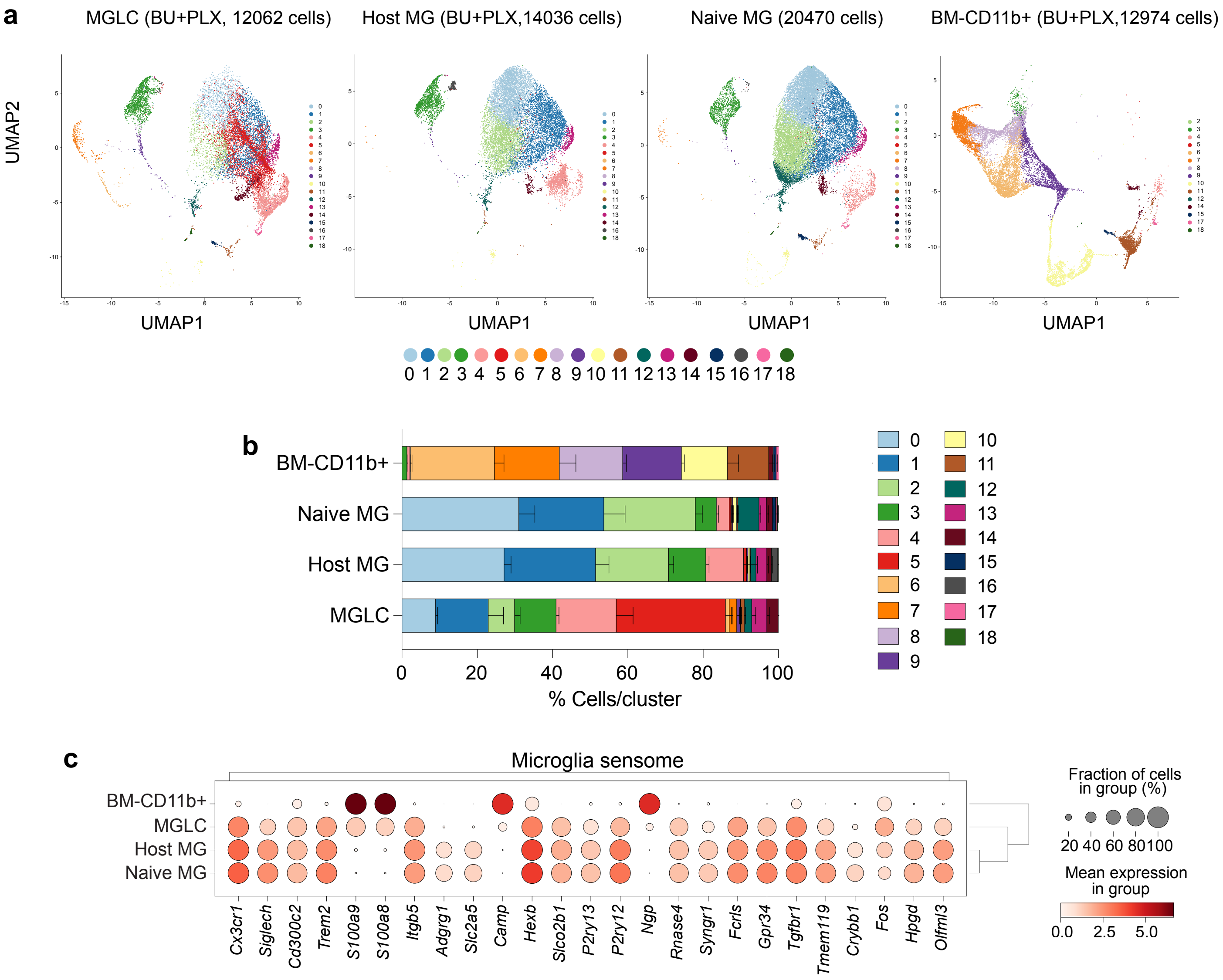

Supplementary Figure 8. Cluster analysis and gene expression profiling of CD45+ CD11b+ cells using single-cell RNA sequencing.

a-c Analyses of single-cell RNA sequencing (scRNA-seq) data performed on FACS-sorted CD45+CD11b+ cells isolated from adult C57BL/6 mice conditioned with busulfan + PLX3397 (BU+PLX) and transplanted with hematopoietic stem and progenitor cells (HSPCs) isolated from C57BL/6-CAG-GFP mice and expanded in culture. The time point of the analyses is 9 months (day 270) after transplant. GFP+ CD45+ CD11b+ (microglia-like cells or MGLCs, n = 3 mice), GFP- CD45+ CD11b+ (host conditioned MG or host MG, n = 3 mice), GFP+ CD45+ CD11b+ from bone marrow (BM-CD11b+, n = 3 mice). Age- and sex-matched untreated mice were used as control (naive MG, n = 3 mice). The FACS sorting scheme is depicted in **Fig. 4a**; the chimerism data are reported in **Supplementary Fig. 4**. **a** Seurat cluster analyses of MGLCs, host-MG, naive MG, and BM-CD11b+ cells. Each cluster is indicated by a color and a number (n = 3 mice/group). The total number of cells analyzed/group is depicted. **b** Comparison of cluster abundance in each sample (n = 3 mice/sample). The percentage of cells contained in each cluster is depicted. Data are Mean ± SD. Source data are provided as a Source Data file. **c** Dot plot showing the differential gene expression of microglia sensome signature genes (Hickman et al.[82]) in each sample (n = 3 mice/sample). The dot size indicates the percentage of cells expressing the gene in each sample/cluster, while the color scale represents the mean gene expression, corresponding to the mean log-normalized UMI counts for each gene. Dendrograms at the right show the sample clustering.

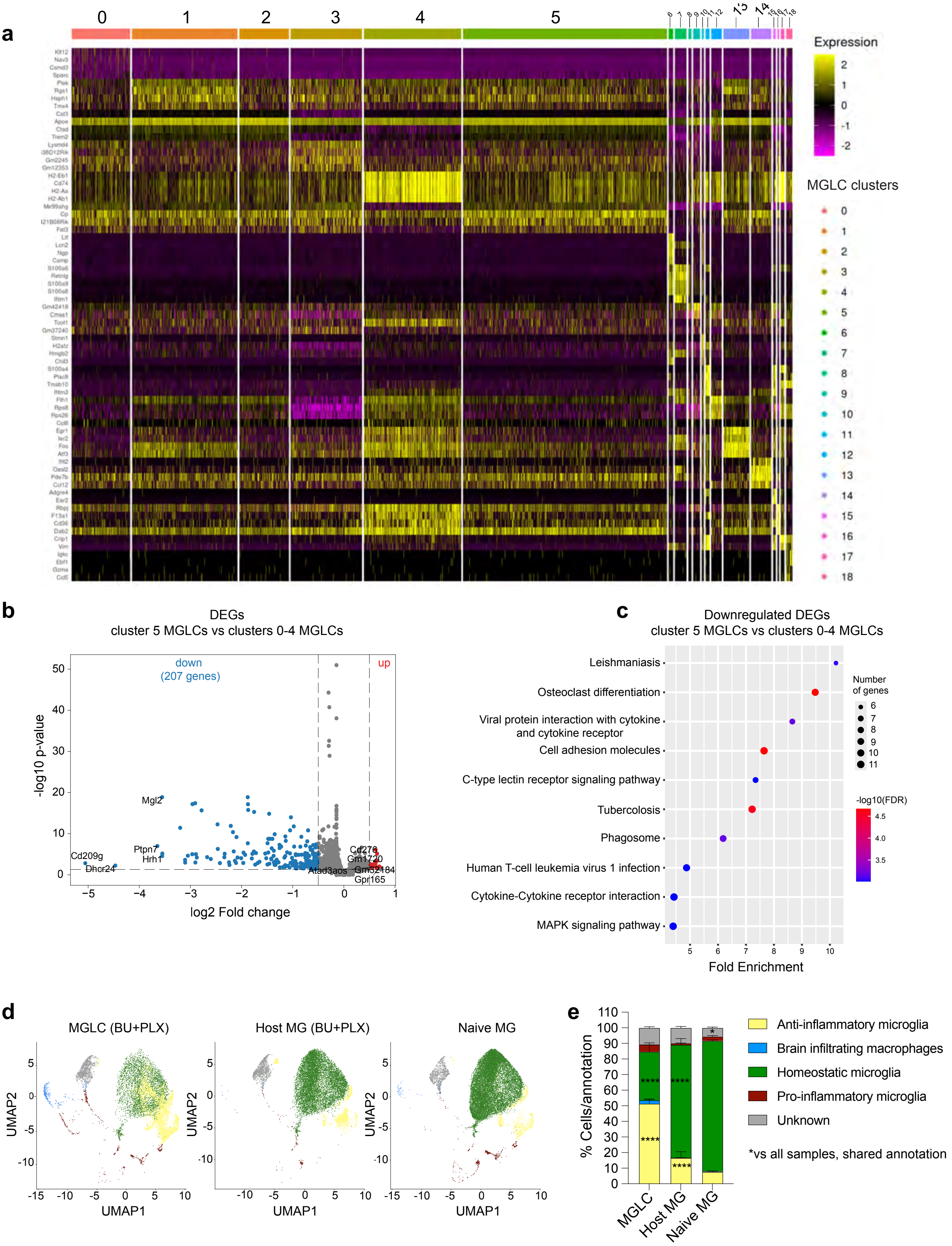
a
Expression
MGLC clusters
b
DEGs
cluster 5 MGLCs vs clusters 0-4 MGLCs
down
(207 genes)
up
-log10 p-value
log2 Fold change
c
Downregulated DEGs
cluster 5 MGLCs vs clusters 0-4 MGLCs
Leishmaniasis
Osteoclast differentiation
Viral protein interaction with cytokine and cytokine receptor
Cell adhesion molecules
C-type lectin receptor signaling pathway
Tubercolosis
Phagosome
Human T-cell leukemia virus 1 infection
Cytokine-Cytokine receptor interaction
MAPK signaling pathway
Number of genes
-log10(FDR)
Fold Enrichment
d
MGLC (BU+PLX)
Host MG (BU+PLX)
Naive MG
UMAP1
UMAP2
e
% Cells/annotation
MGLC
Host MG
Naive MG
Anti-inflammatory microglia
Brain infiltrating macrophages
Homeostatic microglia
Pro-inflammatory microglia
Unknown
*vs all samples, shared annotation

Supplementary Figure 9. Gene expression profiling of CD45+ CD11b+ cells using single-cell RNA sequencing.

a-c Analyses of single-cell RNA sequencing (scRNA-seq) data performed on FACS-sorted CD45+CD11b+ cells isolated from adult C57BL/6 mice conditioned with busulfan + PLX3397 (BU+PLX) and transplanted with hematopoietic stem and progenitor cells (HSPCs) isolated from adult C57BL/6-CAG-GFP mice and expanded in culture. The time point of the analyses is 9 months (day 270) after transplant. GFP+ CD45+ CD11b+ (microglia-like cells or MGLCs, n = 3 mice), GFP- CD45+ CD11b+ (host conditioned MG or host MG, n = 3 mice), GFP+ CD45+ CD11b+ from bone marrow (BM-CD11b+, n = 3 mice). Age- and sex-matched untreated mice were used as control (naive MG, n = 3 mice). The FACS sorting scheme is depicted in **Fig. 4a**; the chimerism data are reported in **Supplementary Fig. 4**. **a** Heat map showing the top 10 differentially expressed genes (DEGs) per cluster of MGLCs (n = 3 mice); expression scale = z-score. Some genes are included in the top set of multiple clusters. Each column in the heat map depicts the expression of a single cell. The bar color indicates the clusters. **b** Volcano plot showing the differentially expressed genes (DEGs) in MGLC cluster 5 vs MGLC clusters 0 to 4 (n=3 mice/cluster). The top 5 downregulated and upregulated gene names are indicated (x-axis: mean log2 fold change; y-axis statistical significance, -log10 adjusted p-value). **c** Pathway enrichment analyses based on the DEGs downregulated in MGLC cluster 5 vs MGLC clusters 0 to 4 (n=3 mice/cluster). The top 10 significantly enriched pathways and the Fold enrichment are depicted. The dot size reflects the numbers of deregulated genes in each pathway, the color scale indicates the -log10 False Discovery Rate (FDR). FDR lower than 0.05 is considered statistically significant. **d** Uniform Manifold Approximation and Projection for Dimension Reduction (UMAP) showing the annotation of cells in each sample as homeostatic microglia, pro-inflammatory microglia, brain infiltrating macrophages, anti-inflammatory microglia or not annotated (unknown), n = 3 mice/sample. **e** Percentage of cells included in each annotation. Data are Mean ± SD. Source data are provided as a Source Data file. Statistical analysis: *p < 0.05, ****p < 0.0001, the exact p-values of all comparisons are reported in the Source Data file; **b** Wald with Benjamini-Hochberg post-hoc correction; **e** Two-way ANOVA with Tukey post-hoc.

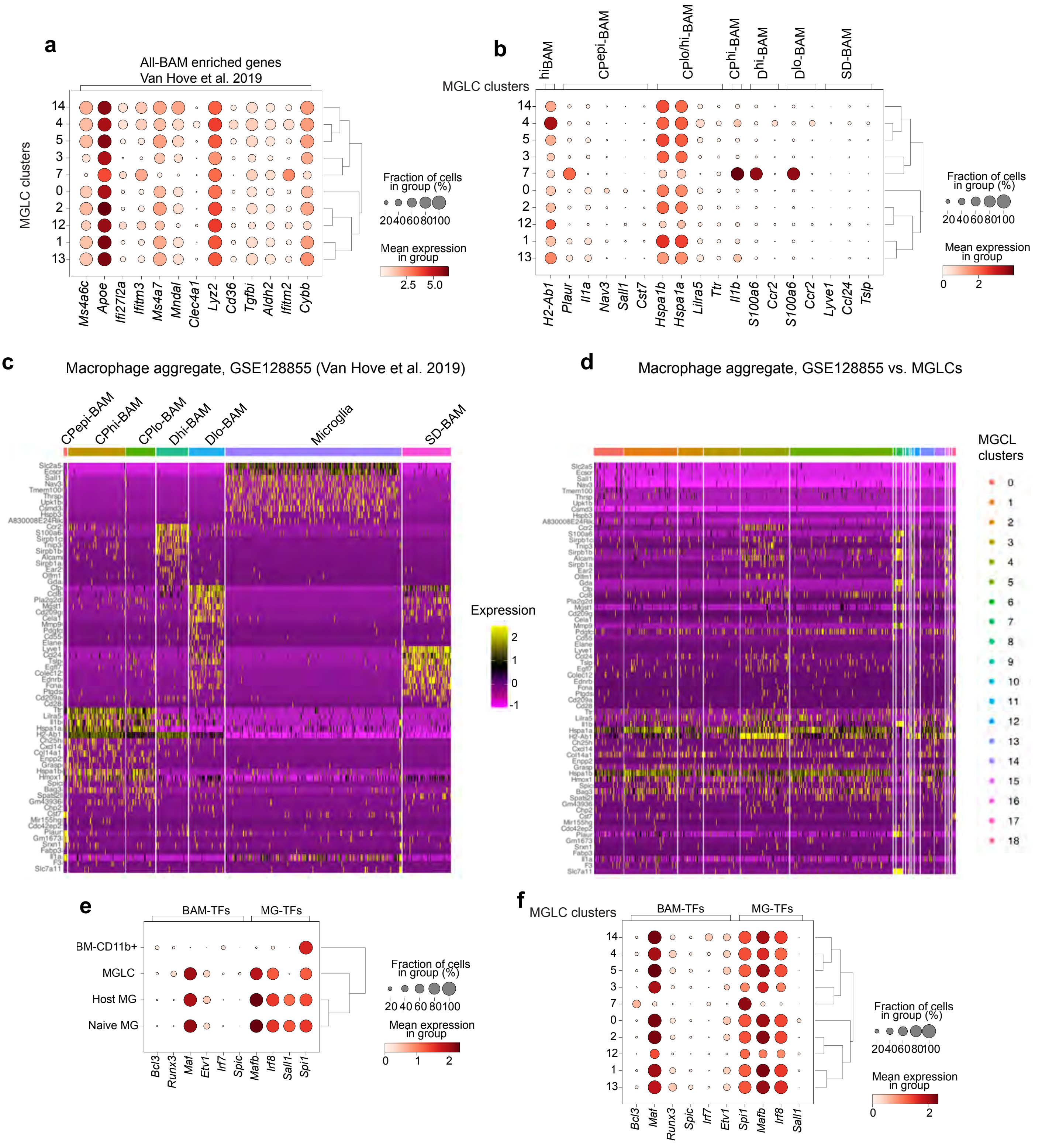
a
All-BAM enriched genes
Van Hove et al. 2019
MGLC clusters
14
4
5
3
7
0
2
12
1
13
Ms4a6c
Apoe
Ifi27l2a
Ifitm3
Ms4a7
Mndal
Clec4a1
Lyz2
Cd36
Tgfbi
Aldh2
Ifitm2
Cybb
Fraction of cells in group (%)
20 40 60 80 100
Mean expression in group
2.5 5.0
b
MGLC clusters
hiBAM
CPepi-BAM
CPlo/hi-BAM
CPhi-BAM
Dhi-BAM
Dlo-BAM
SD-BAM
H2-Ab1
Plaur
Il1a
Nav3
Sall1
Cst7
Hspa1b
Hspa1a
Lilra5
Ttr
Il1b
S100a6
Ccr2
S100a6
Ccr2
Lyve1
Ccl24
Tslp
Fraction of cells in group (%)
20 40 60 80 100
Mean expression in group
0 2
c
Macrophage aggregate, GSE128855 (Van Hove et al. 2019)
CPepi-BAM
CPhi-BAM
CPlo-BAM
Dhi-BAM
Dlo-BAM
Microglia
SD-BAM
Expression
2
1
0
-1
d
Macrophage aggregate, GSE128855 vs. MGLCs
MGCL clusters
0
1
2
3
4
5
6
7
8
9
10
11
12
13
14
15
16
17
18
e
BAM-TFs
MG-TFs
BM-CD11b+
MGLC
Host MG
Naive MG
Bcl3
Runx3
Maf
Etv1
Irf7
Spic
Mafb
Irf8
Sall1
Spi1
Fraction of cells in group (%)
20 40 60 80 100
Mean expression in group
0 1 2
f
MGLC clusters
BAM-TFs
MG-TFs
14
4
5
3
7
0
2
12
1
13
Bcl3
Maf
Runx3
Spic
Irf7
Etv1
Spi1
Mafb
Irf8
Sall1
Fraction of cells in group (%)
20 40 60 80 100
Mean expression in group
0 2

Supplementary Figure 10. MGLCs upregulate genes characteristic of microglia and brain-border-associated macrophages (BAM).

a-b Analyses of single-cell RNA sequencing (scRNA-seq) data performed on FACS-sorted CD45+CD11b+ cells isolated from adult C57BL/6 mice conditioned with busulfan + PLX3397 (BU+PLX) and transplanted with hematopoietic stem and progenitor cells (HSPCs) isolated from adult C57BL/6-CAG-GFP mice and expanded in culture. The time point of the analyses is 9 months (day 270) after transplant. GFP+ CD45+ CD11b+ (microglia-like cells or MGLCs, n = 3 mice), GFP- CD45+ CD11b+ (host conditioned MG or host MG, n = 3 mice), GFP+ CD45+ CD11b+ from bone marrow (BM-CD11b+, n = 3 mice). Age- and sex-matched untreated mice were used as control (naive MG, n = 3 mice). The FACS sorting scheme is depicted in **Fig. 4a**; the chimerism data are reported in **Supplementary Fig. 4**. **a** Dot plot showing the expression of BAM-enriched signature genes, reported by Van Hove et al (GSE128855)[86], in the MGLC clusters from the present study (GSE261246, n=3 mice/cluster). **b** Dot plot showing the expression of selected top differentially expressed genes (DEGs) among BAM subpopulations (panel **c**) in MGLC clusters from the present study (GSE261246, n=3 mice/cluster). Dura BAM: D-BAM; subdural meninges BAM: SD-BAM; choroid plexus BAM: CP-BAM. Major histocompatibility complex II (MHCII) genes such as *H2-Ab1,* distinguish BAM expressing high (^{high}BAM) and low (^{low}BAM) levels of MHCII[86]. **c** Heat map showing the top ten DEGs in D-BAM, SD-BAM, CP-BAM computed using the single-cell RNA-seq data generated by Van Hove et al. (Macrophage aggregate, GSE128855)[86]. **d** Projection of the top 10 DEGs identified in D-BAM, SD-BAM, and CP-BAM (panel **c**) on the MGLC subpopulations/clusters identified in the present work (GSE261246, n = 3 mice/cluster). **c-d** the expression scale indicates the z-score. Some genes are included in the top set of multiple clusters. Each column in the heat map depicts the expression of a single cell. The bar color indicates the subpopulation/clusters. **e-f** Dot plot showing the expression of master transcription factors of microglia and BAMs in each sample (**e**) or MGLC clusters (**f**). Dot size: percentage of cells expressing the gene in each sample/cluster; color scale: mean gene expression, corresponding to the mean log-normalized UMI counts for each gene of interest. Dendrograms at the right show the sample clustering. Source data are provided as a Source Data file.

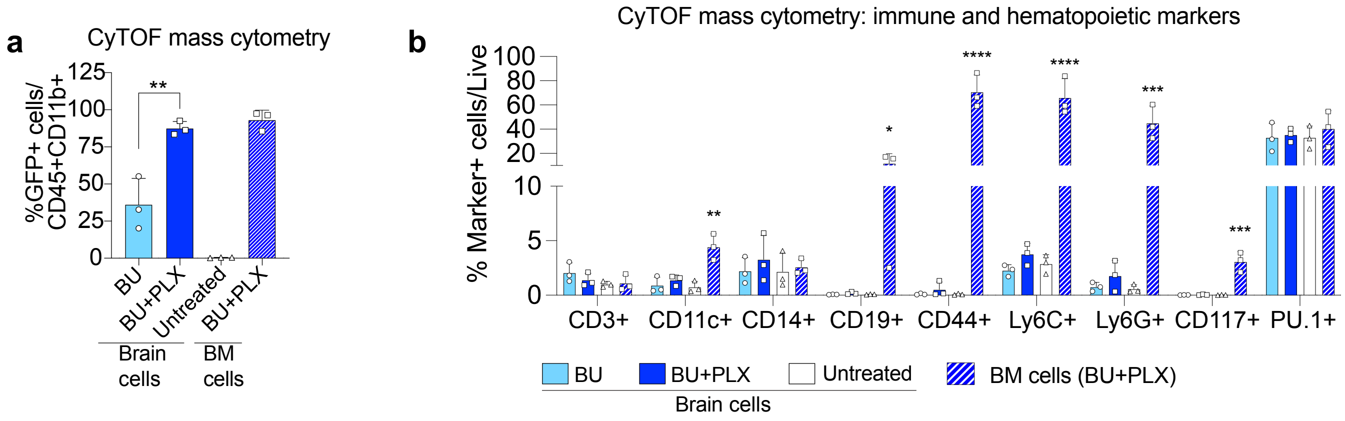

Supplementary Figure 11. Engraftment of MGLCs and analyses of immune cell markers measured by high-dimensional CyTOF mass cytometry.

a-b Analyses of marker expression by high-dimensional CyTOF mass cytometry in cells isolated from the brain and bone marrow of adult C57BL/6 mice conditioned with either busulfan (BU, n = 3 mice) or BU + PLX3397 (BU+PLX, n = 3 mice) and transplanted with bone marrow from adult C57BL/6-CAG-GFP mice (study depicted in **Fig. 1**). The time point of the analyses is 7 months after bone marrow transplant (BMT). **a** Percentage of transplant-derived GFP+ out of total CD45+CD11b+ as measured by anti-GFP intracellular staining in brain and bone marrow. **b** Percentage of marker-positive cells relative to the total live cells isolated from the brain or bone marrow in each treatment group. The individual markers are depicted on the x-axis. **a-b** Data are reported as Mean ± SD. Source data are provided as a Source Data file. Statistical analyses: *p < 0.05, **p < 0.01, ***p < 0.001, ****p < 0.0001, the exact p-values of all comparisons are reported in the Source Data file; **a** Two-tailed unpaired t-test between BU vs. BU+PLX (brain); **b** One-way ANOVA vs. with Tukey post-hoc for each marker.

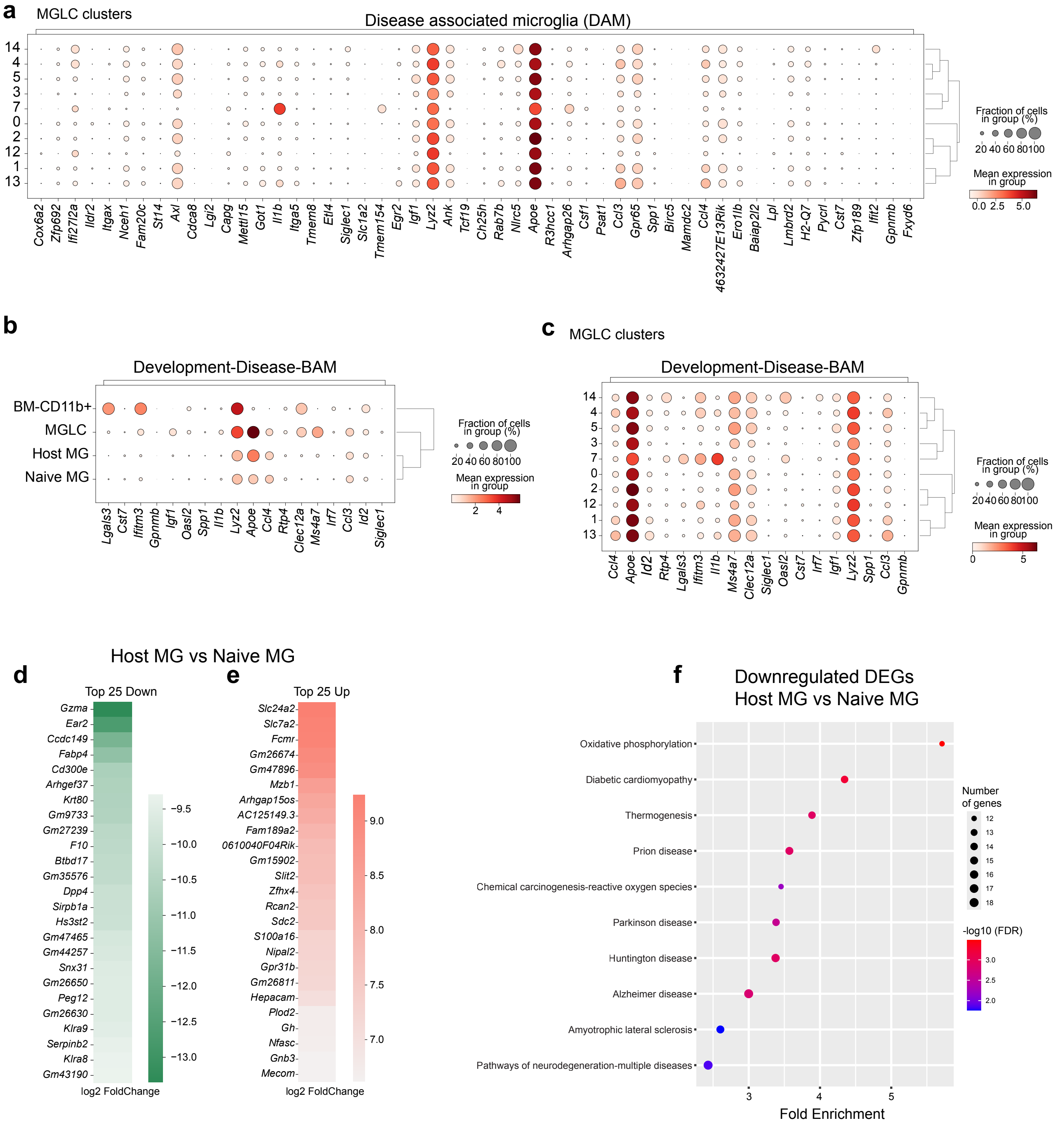
a
MGLC clusters
Disease associated microglia (DAM)
14
4
5
3
7
0
2
12
1
13
Cox6a2 Zfp692 Ifi27l2a Ildr2 Itgax Nceh1 Fam20c St14 Axl Cdca8 Lgi2 Capg Mettl15 Got1 Il1b Itga5 Tmem8 Etl4 Siglec1 Slc1a2 Tmem154 Egr2 Igf1 Lyz2 Ank Tcf19 Ch25h Rab7b Nlrc5 Apoe R3hcc1 Arhgap26 Csf1 Psat1 Ccl3 Gpr65 Spp1 Birc5 Mamdc2 Ccl4 4632427E13Rik Ero1lb Baiap2l2 Lpl Lmbrd2 H2-Q7 Pycrl Cst7 Zfp189 Ifit2 Gpnmb Fxyd6
Fraction of cells in group (%)
20 40 60 80 100
Mean expression in group
0.0 2.5 5.0
b
Development-Disease-BAM
BM-CD11b+
MGLC
Host MG
Naive MG
Lgals3 Cst7 Ifitm3 Gpnmb Igf1 Oasl2 Spp1 Il1b Lyz2 Apoe Ccl4 Rtp4 Clec12a Ms4a7 Irf7 Ccl3 Id2 Siglec1
Fraction of cells in group (%)
20 40 60 80 100
Mean expression in group
2 4
c
MGLC clusters
Development-Disease-BAM
14
4
5
3
7
0
2
12
1
13
Ccl4 Apoe Id2 Rtp4 Lgals3 Ifitm3 Il1b Ms4a7 Clec12a Siglec1 Oasl2 Cst7 Irf7 Igf1 Lyz2 Spp1 Ccl3 Gpnmb
Fraction of cells in group (%)
20 40 60 80 100
Mean expression in group
0 5
Host MG vs Naive MG
d
Top 25 Down
Gzma
Ear2
Ccdc149
Fabp4
Cd300e
Arhgef37
Krt80
Gm9733
Gm27239
F10
Btbd17
Gm35576
Dpp4
Sirpb1a
Hs3st2
Gm47465
Gm44257
Snx31
Gm26650
Peg12
Gm26630
Klra9
Serpinb2
Klra8
Gm43190
−9.5
−10.0
−10.5
−11.0
−11.5
−12.0
−12.5
−13.0
log2 FoldChange
e
Top 25 Up
Slc24a2
Slc7a2
Fcmr
Gm26674
Gm47896
Mzb1
Arhgap15os
AC125149.3
Fam189a2
0610040F04Rik
Gm15902
Slit2
Zfhx4
Rcan2
Sdc2
S100a16
Nipal2
Gpr31b
Gm26811
Hepacam
Plod2
Gh
Nfasc
Gnb3
Mecom
9.0
8.5
8.0
7.5
7.0
log2 FoldChange
f
Downregulated DEGs
Host MG vs Naive MG
Oxidative phosphorylation
Diabetic cardiomyopathy
Thermogenesis
Prion disease
Chemical carcinogenesis-reactive oxygen species
Parkinson disease
Huntington disease
Alzheimer disease
Amyotrophic lateral sclerosis
Pathways of neurodegeneration-multiple diseases
3
4
5
Fold Enrichment
Number of genes
12
13
14
15
16
17
18
-log10 (FDR)
3.0
2.5
2.0

Supplementary Figure 12. Expression of disease-development-border-associated macrophage genes in MGLCs and altered gene expression in host microglia.

a-f Analyses of single-cell RNA sequencing (scRNA-seq) data performed on FACS-sorted CD45+CD11b+ cells isolated from adult C57BL/6 mice conditioned with busulfan + PLX3397 (BU+PLX) and transplanted with hematopoietic stem and progenitor cells (HSPCs) isolated from adult C57BL/6-CAG-GFP mice and expanded in culture. The time point of the analyses is 9 months (day 270) after transplant. GFP+ CD45+ CD11b+ (microglia-like cells or MGLCs, n = 3 mice), GFP- CD45+ CD11b+ (host conditioned MG or host MG, n = 3 mice), GFP+ CD45+ CD11b+ from bone marrow (BM-CD11b+, n = 3 mice). Age- and sex-matched untreated mice were used as control (naive MG, n = 3 mice). The FACS sorting scheme is depicted in **Fig. 4a**; the chimerism data are reported in **Supplementary Fig. 4**. **a** Dot plot showing the levels of expression of disease-associated microglia (DAM) genes (≥ 3-fold upregulation in DAM vs. naive microglia, Keren-Shaul, et al. 2017[92]) in the MGLC clusters. **b-c** Dot plot showing the expression of genes reported being upregulated by microglia during disease, development, and in BAMs. The expression of disease-development-BAM genes is evaluated in each sample (**b**) and in MGLC clusters (**c**). **a-c** Dot size: percentage of cells expressing the gene in each sample/cluster; color scale: mean gene expression, corresponding to the mean log-normalized UMI counts for each gene of interest. Dendrograms at the right show the sample clustering. **d-e** Top 25 differentially expressed genes (DEGs) in conditioned host MG vs naive MG either downregulated (**d**) or upregulated (**e**). The log2 fold change is reported. **f** Pathway enrichment analyses using the downregulated DEGs in host MG vs naive MG. The top 10 significantly enriched pathways are depicted. The dot size reflects the number of genes identified in each pathway while the color scale indicates the -log10 False discovery rate (FDR). FDR lower than 0.05 is considered statistically significant. Statistical analysis: **d-e** Wald with Benjamini-Hochberg post-hoc correction; the exact p-values of all comparisons are reported in the Source Data file. Source data are provided as a Source Data file.

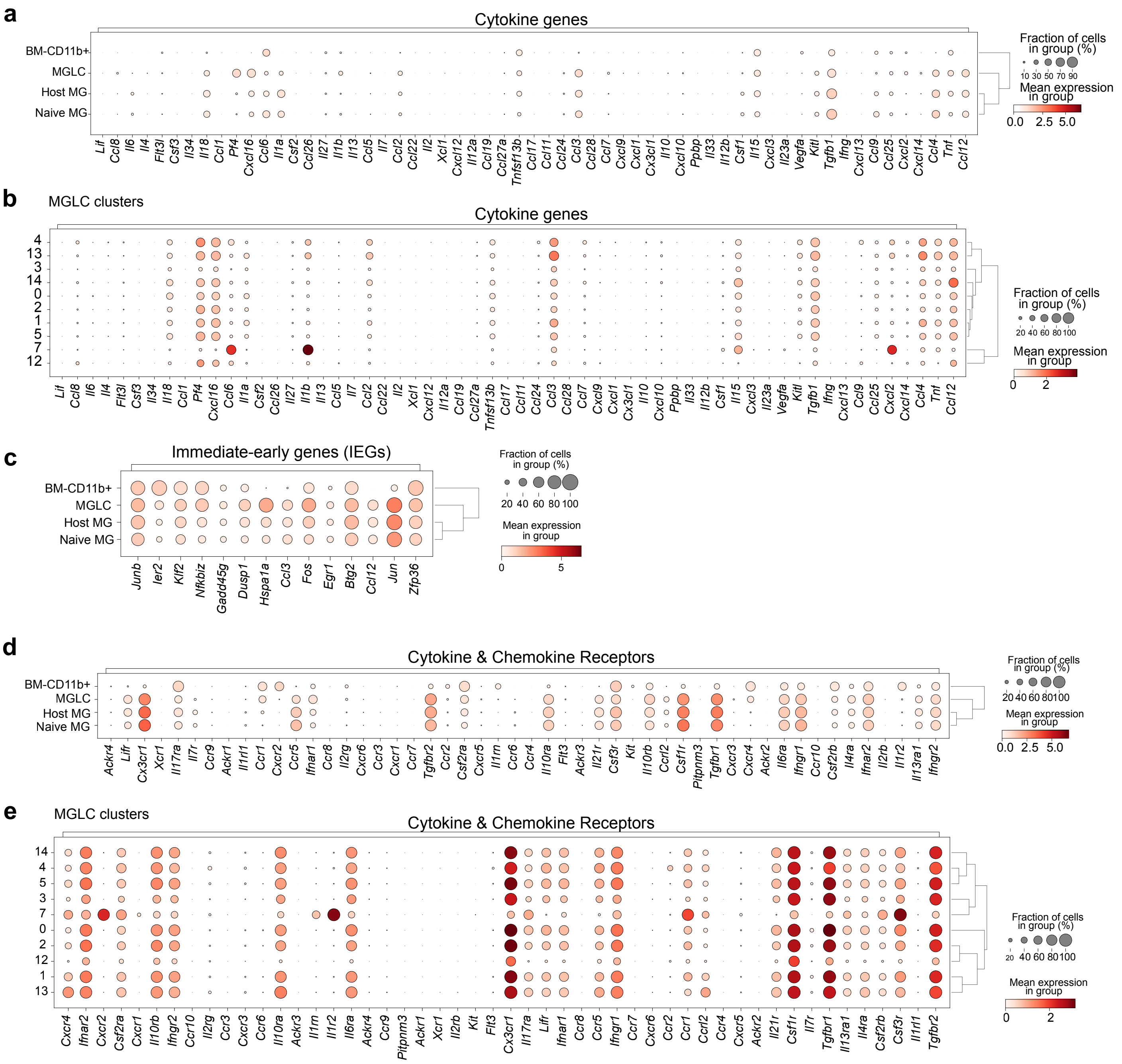

Supplementary Figure 13. Differential expression of cytokine genes, cytokine receptors genes, and immediate-early genes.
a-e Analyses of single-cell RNA sequencing (scRNA-seq) data performed on FACS-sorted CD45+CD11b+ cells isolated from adult C57BL/6 mice conditioned with busulfan + PLX3397 (BU+PLX) and transplanted with hematopoietic stem and progenitor cells (HSPCs) isolated from adult C57BL/6-CAG-GFP mice and expanded in culture. The time point of the analyses is 9 months (day 270) after transplant. GFP+ CD45+ CD11b+ (microglia-like cells or MGLCs, n = 3 mice), GFP- CD45+ CD11b+ (host conditioned MG or host MG, n = 3 mice), GFP+ CD45+ CD11b+ from bone marrow (BM-CD11b+, n = 3 mice). Age- and sex-matched untreated mice were used as control (naive MG, n = 3 mice). The FACS sorting scheme is depicted in **Fig. 4a**; the chimerism data are reported in **Supplementary Fig. 4**. **a-b** Dot plot showing the expression of cytokine genes in each sample (**a**) and in MGLC cluster (**b**). **c** Dot plot showing the expression of immediate-early genes (IEGs) in each sample. IEGs are reported to be induced following enzymatic microglia isolation. **d-e** Dot plot showing the expression of cytokine and chemokine receptor genes in each sample (**d**) and in each MGLC cluster (**e**). **a-e** Dot size: percentage of cells expressing the gene in each sample/cluster; color scale: mean gene expression, corresponding to the mean log-normalized UMI counts for each gene of interest. Dendrograms at the right show the sample clustering. Source data are provided as a Source Data file.

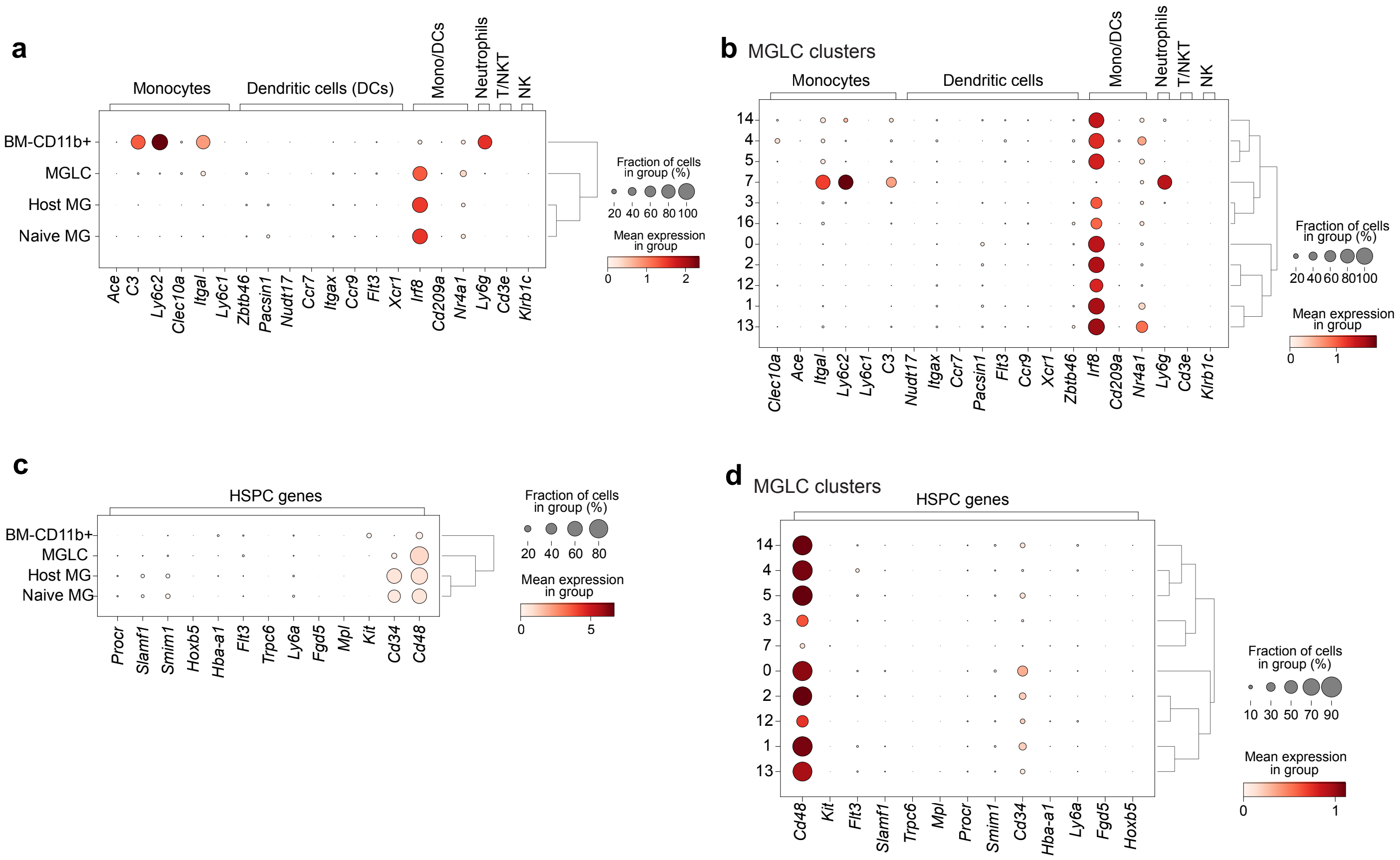

Supplementary Figure 14. Differential expression of genes expressed in hematopoietic lineages: monocytes, dendritic cells, lymphocytes, and hematopoietic stem and progenitor cells.
a-d Analyses of single-cell RNA sequencing (scRNA-seq) data performed on FACS-sorted CD45+CD11b+ cells isolated from adult C57BL/6 mice conditioned with busulfan + PLX3397 (BU+PLX) and transplanted with hematopoietic stem and progenitor cells (HSPCs) isolated from adult C57BL/6-CAG-GFP mice and expanded in culture. The time point of the analyses is 9 months (day 270) after transplant. GFP+ CD45+ CD11b+ (microglia-like cells or MGLCs, n = 3 mice), GFP- CD45+ CD11b+ (host conditioned MG or host MG, n = 3 mice), GFP+ CD45+ CD11b+ from bone marrow (BM-CD11b+, n = 3 mice). Age- and sex-matched untreated mice were used as control (naive MG, n = 3 mice). The FACS sorting scheme is depicted in **Fig. 4a**; the chimerism data are reported in **Supplementary Fig. 4**. **a-b** Dot plot showing the expression of genes characteristic of monocytes, dendritic cells, neutrophils, T lymphocytes, and natural killer (NK) cells in each sample (**a**) and in MGLC clusters (**b**). **c-d** Dot plot showing the expression of genes characteristic of hematopoietic stem and progenitor cells (HSPCs) in each sample (**c**) and in MGLC clusters (**d**). HSPC markers include *Ly6a* (SCA-1), *Kit* (KIT/CD117), *Slamf1* (CD150) and *Procr* (EPCR). **a-d** Dot size: percentage of cells expressing the gene in each sample/cluster; color scale: mean gene expression, corresponding to the mean log-normalized UMI counts for each gene of interest. Dendrograms at the right show the sample clustering. Source data are provided as a Source Data file.

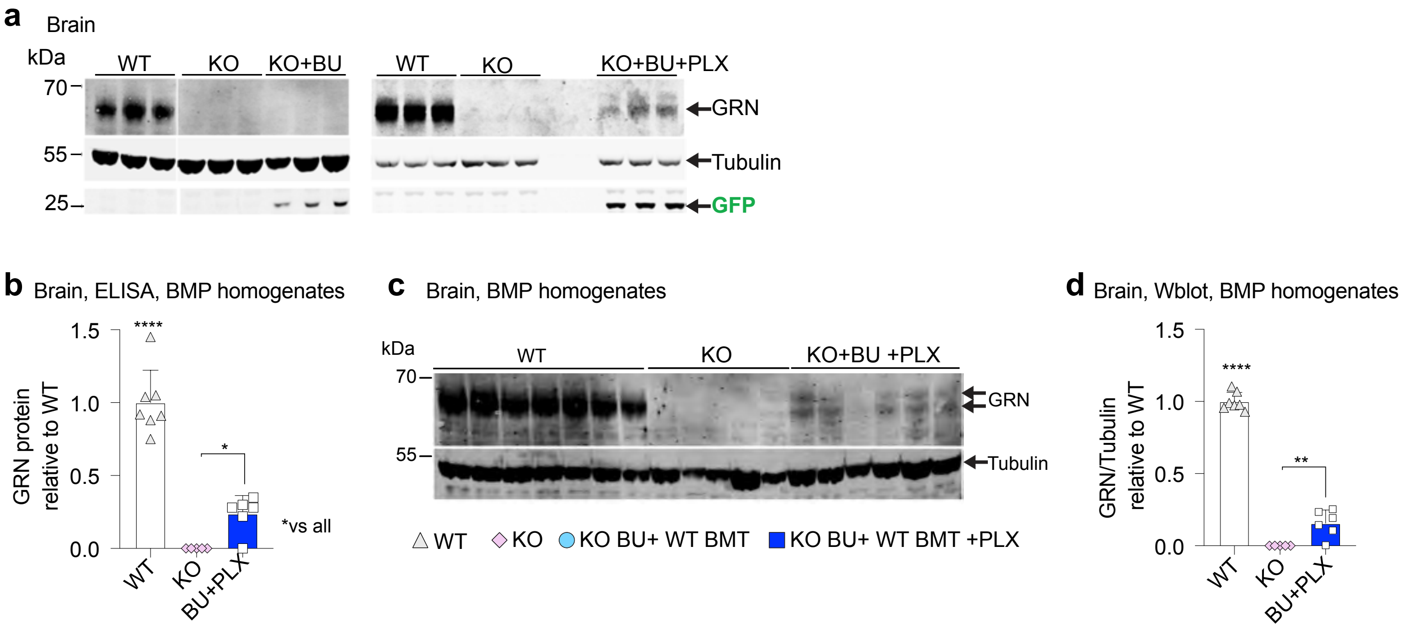

Supplementary Figure 15. Microglia replacement corrects progranulin deficiency in a mouse model of CLN11/FTD following wild-type bone marrow transplant and busulfan plus PLX3397 conditioning.
a Analysis of 6-month-old *Grn-/-* (KO) mice and WT controls. The study scheme and timeline are depicted in **Fig. 7c**. Analyses 4 months after bone marrow transplant (BMT). Follow-up was 4 months (D120) after transplant. Healthy controls: age-matched wild-type C57BL/6 mice (WT); disease controls: KO mice. **a** Representative Western blot analysis of GRN protein amounts in mouse brain lysates at the study end. Tubulin was used as a loading control; the anti-GFP antibody was used to detect the GFP protein expressed by transplant-derived cells. Uncropped blots are in Source Data and Supplementary Fig. 17. The blot is representative of the following mice: n = 6 WT, n=5 KO, n = 6 BU, n=6 BU+PLX. **b** Quantification of GRN protein by ELISA assay in brain homogenates used for the quantification of Bis(Monoacylglycero)Phosphate (BMP) lipids and depicted in **Fig. 8g-h**; mouse numbers (n): WT n = 7, KO n = 5, BU+PLX n = 6). **c-d** Quantification of GRN protein by Western Blots in brain homogenates used for the quantification of BMP lipids and depicted in **Fig. 8g-h. c** Western Blot depicting GRN amounts in the brain homogenates; tubulin was used as a loading control; the uncropped blot is depicted in panel c; **d** Quantification of GRN band intensities normalized by tubulin . **c-d** mouse numbers (n): WT n = 7, KO n = 5, BU+PLX n = 6. **b, d** Data are Mean ± SD. The quantification is relative to WT mice. Source data are provided as a Source Data file. Statistical analysis: *p < 0.05, **p < 0.01, ****p < 0.0001, the exact p-values of all comparisons are reported in the Source Data file; **b** Kruskal-Wallis with Holm-Sidak post-hoc; **d** One-way ANOVA with Tukey post-hoc.

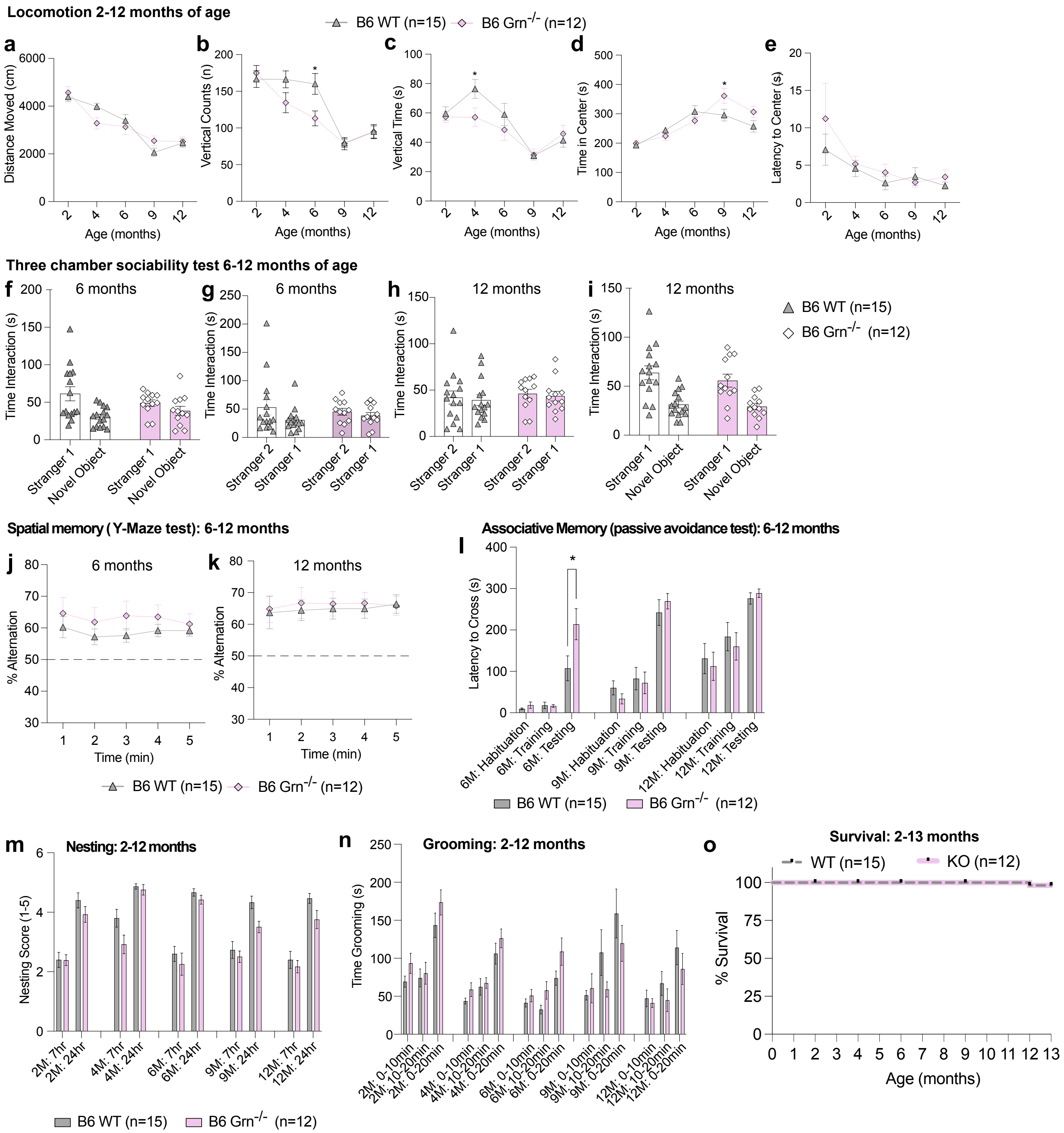

Supplementary Figure 16. Serial neurobehavioral phenotyping and survival of *Grn$^{-/-}$* mice.
a-n Neurobehavioral analysis of untreated wild-type (WT) and untreated *Grn$^{-/-}$* (KO) male mice from 2 to 12 months of age. **a-e** Analyses of mouse locomotion and exploratory behavior at 2, 4, 6, 9, and 12 months of age; mouse numbers (n): WT n =15, KO n =13 at 2 months, KO n=12 at 4, 6, 9, and 12 months. **f-i** Analyses of mouse sociability evaluated using the 3-chamber sociability test at 6 and 12 months of age; mouse numbers (n): WT n=15, KO n=12. **j-k** Analyses of mouse spatial memory using the Y-maze spontaneous alternation test at 6 and 12 months of age; mouse numbers (n): WT n=15, KO n=12. **l** Analyses of mouse associative memory using the passive avoidance test, at 6, 9, and 12 months of age; mouse numbers (n): WT n=15, KO n=12 at 6 and 9 months; WT n=14, KO n=11 at 12 months. **m** Analyses of mouse cognitive deficit evaluated by their nesting ability at 2, 4, 6, 9, and 12 months of age; mouse numbers (n): WT n=15, KO n=13 at 2 months, KO n=12 at 4, 6, 9, and 12 months. **n** Analyses of mouse obsessive-compulsive behavior, reflected by their grooming activity at 2, 4, 6, 9, and 12 months of age; mouse numbers (n): WT n=15, KO n=12. **a-n** Data are reported as Mean ± SE. Source data are provided as a Source Data file. Statistical analyses: *p < 0.05, **p < 0.01, ***p < 0.001, ****p < 0.0001, the exact p-values of all comparisons are reported in the Source Data file; **a-n** Two-way ANOVA with Sidak post-hoc. **o** Survival curve of WT (n=15) and *Grn$^{-/-}$* (KO, n=12) mice during the neurobehavioral analysis and up to 13 months of age. One mouse in each cohort died at 12.1 months of age.

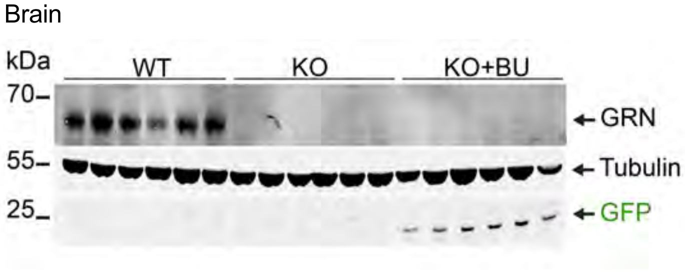

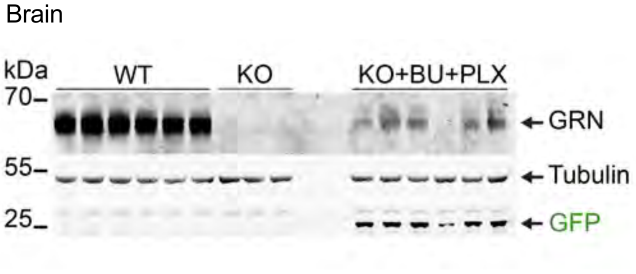

Supplementary Figure 17. Uncropped blots related to Supplementary Figure 15, panel a.
Analysis of brain progranulin (GRN) in 6-month-old *Grn-/-* (KO) mice and WT controls. The study scheme and timeline are depicted in **Fig. 7c**. Uncropped blots of mouse brain lysates at the study end using anti-GRN antibodies. Tubulin was used as a loading control; the anti-GFP antibody was used to detect the GFP protein expressed by transplant-derived cells. Uncropped blots are included in the Source Data file. Mouse numbers (n): n = 6 WT, n = 6 KO, n = 6 BU (left blot); n = 6 WT, n = 3 KO, n = 6 BU n=6 BU+PLX (right blot).

Supplementary Table 1. Number of cells included in the single-cell RNA sequencing analyses.

Sample	MGLC (BU+PLX) number (N) of cells/mouse			Total Cell Number
	Mouse 1	Mouse 2	Mouse 3	
N of cells pre filtering	5684	6325	1452	
N of cells post doublets filtering	5258	5851	1343	
N of cells post nFeature_RNA filtering	5248	5795	1343	
N of cells post mitochondrial filtering	5092	5681	1289	
**N of cells post filtering**	**5092**	**5681**	**1289**	**12062**
**Sample**	**host MG (BU+PLX), number (N) of cells/mouse**			**Total Cell Number**
	**Mouse 1**	**Mouse 2**	**Mouse 3**	
N of cells pre filtering	8535	2596	4346	
N of cells post doublets filtering	7895	2401	4020	
N of cells post nFeature_RNA filtering	7809	2389	4009	
N of cells post mitochondrial filtering	7743	2344	3949	
**N of cells post filtering**	**7743**	**2344**	**3949**	**14036**
**Sample**	**naïve MG, number (N) of cells/mouse**			**Total Cell Number**
	**Mouse 1**	**Mouse 2**	**Mouse 3**	
N of cells pre filtering	8306	7142	6866	
N of cells post doublets filtering	7683	6606	6351	
N of cells post nFeature_RNA filtering	7646	6587	6335	
N of cells post mitochondrial filtering	7603	6559	6308	
**N of cells post filtering**	**7603**	**6559**	**6308**	**20470**
**Sample**	**BM-CD11b+ (BU+PLX), number (N) of cells/mouse**			**Total Cell Number**
	**Mouse 1**	**Mouse 2**	**Mouse 3**	
N of cells pre filtering	4855	4701	4750	
N of cells post doublets filtering	4491	4348	4394	
N of cells post nFeature_RNA filtering	4399	4296	4337	
N of cells post mitochondrial filtering	4375	4278	4321	
**N of cells post filtering**	**4375**	**4278**	**4321**	**12974**

Supplementary Table 2. CyTOF antibody panel.

Antigen	Clone	Supplier	Metal Isotope	Catalog number	RRID	Epitope	Dilution (μg/mL)	Staining[a, b]
CD45	30-F11	Fluidigm	Y89	3089005B	AB_2651 152		1	S
cPARP[c]	F21-852	BD Biosciences	La139	552597	N/A	cleaved	0.5	I
Ly-6G	1A8	Fluidigm	Pr141	3141008B	AB_2814 678		2	S
CD185	614641	Fluidigm	Nd142	3142008B	N/A		2	S
CD115	AFS98	Fluidigm	Nd144	3144012B	AB_2895 116		2	S, I
F4/80	BM8	Fluidigm	Nd146	3146008B	AB_2895 117		2	S
CD11b	M1/70	Fluidigm	Nd148	3148003B	AB_2814 738		2	S
CD19	6D5	Fluidigm	Sm149	3149002B	AB_2814 679		2	S
p53	DO-7	Biolegend	Nd150	3150024B	N/A	total	2	S
CD64	X54-5/7.1	Fluidigm	Eu151	3151012B	AB_2814 680		2	S
Ki67[c]	B56	BD	Sm152	556003	N/A	total	2	I
Mac-2	M3/38	Fluidigm	Eu153	3153026B	AB_2814 900		2	S,I
TER-119	TER-119	Fluidigm	Sm154	3154005B	N/A		2	S
CD14	Sa14-2	Fluidigm	Gd156	3156009B	AB_2814 681		2	S
PU.1[c]	9G7	Cell Signaling Technology	Gd157	22588F	N/A	total	2	I
CD184	L276F12	Fluidigm	Tb159	3159030B	N/A		2	S, I
MIP1beta	D21-1351	Fluidigm	Gd160	3160013B	N/A		2	S,I
Ly-6C	HK1.4	Fluidigm	Dy162	3162014B	AB_2922 921		2	S
CX3CR1	SA011F11	Fluidigm	Dy164	3164023B	AB_2832 247		2	S
CD3e	145-2C11	Fluidigm	Ho165	3165020B	N/A		2	S
GFP	SF12.4	Fluidigm	Tm169	3169009B	AB_2814 899		1	I
CD169	3D6.112	Fluidigm	Er170	3170018B	AB_2885 022		2	S
CD44	IM7	Fluidigm	Yb171	3171003B	AB_2895 121		2	S
CD86	GL1	Fluidigm	Yb172	3172016B	AB_2922 923		2	S
CD117	2B8	Fluidigm	Yb173	3173004B	AB_2811 230		2	S
I-A/I-E	M5/114.15.2	Fluidigm	Yb174	3174003B	AB_2922 924		2	S
prpS6[c]	N7-548	BD Biosciences	Yb175	Custom	N/A	pS235/236	2	I
pCREB	87G3	Fluidigm	Yb176	3176005A	AB_2934 290	pS133	2	I
CD11c	N418	Fluidigm	Bi209	3209005B	AB_2811 244		2	I

[a]Surface staining (S); [b]Intracellular staining (I); [c]Custom conjugation.

Supplementary Table 3. Primary antibodies.

Antigen	Conjugation	Clone	RRID	Supplier	Catalog number	Dilution
BD Fc BloBlock™	unconjugated	2.4G2	AB_394656	BD Biosciences	BDB553142	100
CD45	PE-Cy7	104	AB_2573350	Thermo Fisher Scientific	25-0454-82	100
CD45	PE-Cy7	30F11	AB_312979	Biolegend	103114	100
CD11b	PE	M1/70	AB_312791	BioLegend	101208	100
TER-119	PE-Cy5	TER-119	AB_468810	Thermo Fisher Scientific	15-5921-83	200
Ly6C	BV605	AL-21	AB_2737949	BD Biosciences	BDB563011	100
CD3	APC	17A2	AB_10597589	Thermo Fisher Scientific	17-0032-82	100
CD19	PB	6D3	AB_439718	BioLegend	115523	100
Ly6G	PE	1A8	AB_1186099	Biolegend	127608	100
CD41	PB	MWReg 30	AB_2750526	BioLegend	133932	100
CSF1R	APC	AFS98	AB_1210789	eBioscience	17-1152-82	100
SCA1/Ly-6A/E	PE	D7	AB_466085	Thermo Fisher Scientific	12-5981-81	100
KIT/CD117	APC	2B8	AB_469429	Thermo Fisher Scientific	17-1171-81	100
CD3	PB	17A2	AB_493645	BioLegend	100214	100
TER-119	PB	TER-119	AB_2251160	BioLegend	116232	100
Ly6C	PB	HK1.4	AB_1732079	BioLegend	128014	100
B220	PB	RA3-6B2	AB_492876	BioLegend	103227	100
Progranulin	unconjugated	N/A	AB_2114504	R&D Systems	AF2557-SP	1000
GFP	unconjugated	N/A	AB_221569	Thermo Fisher Scientific	A-11122	1000
Alpha tubulin	unconjugated	DM1A	AB_477583	Sigma-Aldrich	T6199	5000
Cathepsin D	unconjugated	AF1029	AB_2087094	R&D Systems	AF1029	1000
Ubiquitin	unconjugated	N/A	AB_1088148	Thermo Fisher Scientific	PA1-10023	1000
IBA1	unconjugated	N/A	AB_8395040	FUJIFILM Wako Pure Chemical Corporation	19-19741	100
F4/80	unconjugated	BM8.1	AB_2938669	Cell Signaling Technology	71299S	100
TotalSeqTM-B 0301 Hashtag 1 (CD45 and MHCI)	oligo-tagged	30F11 and M1/42	AB_2814067	BioLegend	155831	0.15 µL/ 1x10^6 cells
TotalSeqTM-B 0302 Hashtag 2 (CD45 and MHCI)	oligo-tagged	30F11 and M1/42	AB_2814068	BioLegend	155833	0.15 µL/ 1x10^6 cells
TotalSeqTM-B 0303 Hashtag 3 (CD45 and MHCI)	oligo-tagged	30F11 and M1/42	AB_2814069	BioLegend	155835	0.15 µL/ 1x10^6 cells
