# Supplementary material for: CNS-wide repopulation by hematopoietic-derived microglia-like cells corrects progranulin deficiency in mice
Source: Nat Commun. 2024 Jul 5;15:5654. doi: 10.1038/s41467-024-49908-4 (PMC11226701; doi:10.1038/s41467-024-49908-4)
Supplement: Supplementary file 3 — Reporting Summary [file 41467_2024_49908_MOESM3_ESM.pdf]

nature portfolio

Corresponding author(s): Natalia-Gomez-Ospina, Pasqualina Colella

Last updated by author(s): May 17 2024

Reporting Summary

Nature Portfolio wishes to improve the reproducibility of the work that we publish. This form provides structure for consistency and transparency in reporting. For further information on Nature Portfolio policies, see our Editorial Policies and the Editorial Policy Checklist.

Statistics

For all statistical analyses, confirm that the following items are present in the figure legend, table legend, main text, or Methods section.

n/a	Confirmed	
☐	☒	The exact sample size (*n*) for each experimental group/condition, given as a discrete number and unit of measurement
☐	☒	A statement on whether measurements were taken from distinct samples or whether the same sample was measured repeatedly
☐	☒	The statistical test(s) used AND whether they are one- or two-sided<br>*Only common tests should be described solely by name; describe more complex techniques in the Methods section.*
☒	☐	A description of all covariates tested
☐	☒	A description of any assumptions or corrections, such as tests of normality and adjustment for multiple comparisons
☐	☒	A full description of the statistical parameters including central tendency (e.g. means) or other basic estimates (e.g. regression coefficient) AND variation (e.g. standard deviation) or associated estimates of uncertainty (e.g. confidence intervals)
☒	☐	For null hypothesis testing, the test statistic (e.g. *F*, *t*, *r*) with confidence intervals, effect sizes, degrees of freedom and *P* value noted<br>*Give P values as exact values whenever suitable.*
☒	☐	For Bayesian analysis, information on the choice of priors and Markov chain Monte Carlo settings
☒	☐	For hierarchical and complex designs, identification of the appropriate level for tests and full reporting of outcomes
☒	☐	Estimates of effect sizes (e.g. Cohen's *d*, Pearson's *r*), indicating how they were calculated

Our web collection on statistics for biologists contains articles on many of the points above.

Software and code

Policy information about availability of computer code

Data collection	All softwares for data collection are publicly or commercially available.<br>We did not generate custom computer code in this manuscript.<br><br>Softwares and packages:<br>- CytExpert Software (Beckman Coulter)<br>- MA900 Software (Sony Biotechnology).<br>- Helios instrument control software v7 (Fluidigm)<br>- NovaSeq Control Software v1.8 (Illumina)<br>- BZ-X800 software (Keyence, Itasca).<br>- Mass Hunter v12 software<br>- GIMINI software<br>- Ethovision XT 17.5 (Noldus information technology tracking system)<br>- Activity Monitor Software-811 (Med associates Inc)
Data analysis	All softwares for data analysis are publicly or commercially available.<br>We did not generate custom computer code in this manuscript.<br><br>Softwares and packages:<br>- FlowJo v10.8.2 (FlowJo, LLC)

- Prism v9.5.1 (GraphPad software)
- Microsoft Excel v16.75.2
- OMIQ software from Dotmatics (https://app.omiq.ai)
- Quant-My-Way
- CellRanger software from 10x Genomics (https://support.10xgenomics.com/single-cell-gene-expression/software/overview/welcome)
- Seurat package v4.0
- ScType R package
- Scanpy package v1.9.3
-Decoupler-py v1.5.0
- PyDeSeq2 v0.4.4
- ggplot2 package in R

For manuscripts utilizing custom algorithms or software that are central to the research but not yet described in published literature, software must be made available to editors and reviewers. We strongly encourage code deposition in a community repository (e.g. GitHub). See the Nature Portfolio guidelines for submitting code & software for further information.

Data

Policy information about availability of data

All manuscripts must include a data availability statement. This statement should provide the following information, where applicable:

- Accession codes, unique identifiers, or web links for publicly available datasets
- A description of any restrictions on data availability
- For clinical datasets or third party data, please ensure that the statement adheres to our policy

The data generated in this study are provided in the Source Data file. Single-cell transcriptomic data generated in this study are publicly available in the NCBI's Gene Expression Omnibus (GEO) database, accession number GSE261246 and GSE241877 [https://www.ncbi.nlm.nih.gov/geo/]. Data generated in previous studies and used here as comparison (GSE128855) are publicly available in GEO [https://www.ncbi.nlm.nih.gov/geo/]. Source data are provided with this paper. Supplementary information accompanies this paper. Further correspondence and material requests should be addressed to the corresponding authors Natalia Gomez-Ospina and Pasqualina Colella.

Research involving human participants, their data, or biological material

Policy information about studies with human participants or human data. See also policy information about sex, gender (identity/presentation), and sexual orientation and race, ethnicity and racism.

Reporting on sex and gender	N/A
Reporting on race, ethnicity, or other socially relevant groupings	N/A
Population characteristics	N/A
Recruitment	N/A
Ethics oversight	N/A

Note that full information on the approval of the study protocol must also be provided in the manuscript.

Field-specific reporting

Please select the one below that is the best fit for your research. If you are not sure, read the appropriate sections before making your selection.

☒ Life sciences ☐ Behavioural & social sciences ☐ Ecological, evolutionary & environmental sciences

For a reference copy of the document with all sections, see nature.com/documents/nr-reporting-summary-flat.pdf

Life sciences study design

All studies must disclose on these points even when the disclosure is negative.

Sample size

The sample sizes were chosen based on several factors: Previous Studies: We considered sample sizes used in previous studies. Expected Effect Size: We estimated the expected effect size based on pilot data (Fig. 1b-c) to ensure that the chosen sample sizes would be sufficient to detect meaningful differences if they existed.
The number of sampled units, denoted as "n," represents a single mouse for in vivo experiments (n = 1) or the number of independent biological replicates for in vitro experiments (n = 1). The exact sample size for each experiment is detailed in the corresponding figure legends, with n ≥ 3 for all experiments.

Data exclusions

No data were excluded from analysis in this work.

Replication

The number of sampled units, denoted as "n," represents a single mouse for in vivo experiments (n = 1) or the number of independent biological replicates for in vitro experiments (n = 1). In vivo studies evaluating neurobehavioral changes were conducted with an n of mice > 10. The exact sample size for each experiment is detailed in the corresponding figure legends, with n ≥ 3 for all experiments. All data points are shown for bar chart plots with a sample size less than 10. Information regarding biological replication is included in the figure legends.

The results demonstrating the efficiency of our optimized busulfan+PLX 3397 conditioning at replacing brain microglia with bone marrow-derived microglia-like cells were replicated in seven independent sets of transplantation experiments using C57BL/6 mice. These experiments are detailed as follows: Experiment 1 (Fig. 1a-c), Experiment 2 (Fig. 1d-e/Fig. 3), Experiment 3 (Fig. 2c-d), Experiment 4 (Fig. 4/Supplementary Fig. 4), and Experiment 5 (Supplementary Fig. 1g-i). Additionally, one independent experiment was conducted in C57BL6-Cx3cr1+/- mice (Supplementary Fig. 1j-l; Experiment 6), and one experiment was conducted in C57BL6-Grn-/- mice (Experiment 7).

Randomization

Mice were randomly assigned to each experimental group.

Blinding

Blinding was feasible for neurobehavioral analyses (Fig. 3l-o and Supplementary Fig. 16), histological analyses (Fig. 7 n-o and Supplementary Fig. 3h a), and bioinformatic analyses of scRNA-seq (Fig. 4b-g, 5a-e, 6a-e, Supp. Fig. 8, 9, 10, 12, 13, 14). Neurobehavioral tests were conducted by investigators blinded to the group allocations and/or mouse genotypes. Histological image acquisition and quantification were performed by an investigator blinded to the group allocations. The bioinformatics analyses of the scRNA-seq data were conducted by investigators blinded to the group allocations. Blinding was not feasible for the in vivo experiments involving busulfan+PLX3397 conditioning due to hair pigmentation changes in the treated mice. Unbiased side-by-side processing of samples from different treatment groups was conducted to minimize variability in sample processing.

Reporting for specific materials, systems and methods

We require information from authors about some types of materials, experimental systems and methods used in many studies. Here, indicate whether each material, system or method listed is relevant to your study. If you are not sure if a list item applies to your research, read the appropriate section before selecting a response.

Materials & experimental systems

n/a	Involved in the study	
☐	☒	Antibodies
☒	☐	Eukaryotic cell lines
☒	☐	Palaeontology and archaeology
☐	☒	Animals and other organisms
☒	☐	Clinical data
☒	☐	Dual use research of concern
☒	☐	Plants

Methods

n/a	Involved in the study	
☒	☐	ChIP-seq
☐	☒	Flow cytometry
☒	☐	MRI-based neuroimaging

Antibodies

Antibodies used

All primary antibodies used in this study are described in Supplementary Table 2 and Supplementary Table 3, which includes information regarding epitope, clone, vendor, concentrations, and RRID.

Primary antibodies:

Antigen Conjugation Company name Catalog number Clone RRID Lot
BD Fc BloBlock™ unconjugated BD Biosciences BDB553142 2.4G2 AB_394656 0296888
CD45 PE-Cy7 Thermo Fisher Scientific 25-0454-82 104 AB_2573350 2373751
CD45 PE-Cy7 Biolegend 103114 30F11 AB_312979 B354212
CD11b PE BioLegend 101208 M1/70 AB_312791 B357279
TER-119 PE-Cy5 Thermo Fisher Scientific 15-5921-83 TER-119 "
AB_468810" 2452835
Ly6C BV605 BD Biosciences BDB563011 AL-21 AB_2737949 3044051
CD3 APC Thermo Fisher Scientific 17-0032-82 17A2 "
AB_10597589" 2400617
CD19 PB BioLegend 115523 6D3 AB_439718 B355147
Ly6G PE Biolegend 127608 1A8 AB_1186099 B353768
CD41 PB BioLegend 133932 MWReg30 AB_2750526 B300583
CSF1R APC eBioscience 17-1152-82 AFS98 AB_1210789 2111799
SCA1/Ly-6A/E PE Thermo Fisher Scientific 12-5981-81 D7 AB_466085 2171950
KIT/CD117 APC Thermo Fisher Scientific 17-1171-81 2B8 "
AB_469429" 2660440
CD3 PB BioLegend 100214 17A2 AB_493645 B363201
TER-119 PB BioLegend 116232 TER-119 AB_2251160 B357693

Ly6C PB BioLegend 128014 HK1.4 AB_1732079 B360197
B220 PB BioLegend 103227 RA3-6B2 AB_492876 B375501
Progranulin unconjugated R&D Systems AF2557-SP N/A AB_2114504 UWR0122051
GFP unconjugated Thermo Fisher Scientific A-11122 N/A AB_221569 2339829
Alpha tubulin unconjugated Sigma-Aldrich T6199 DM1A AB_477583 Not Avialable
Cathepsin D unconjugated R&D Systems AF1029 AF1029 AB_2087094 GZPO323071
Ubiquitin unconjugated Thermo Fisher Scientific PA1-10023 N/A AB_1088148 Not Avialable
IBA1 unconjugated FUJIFILM Wako Pure Chemical Corporation 19-19741 N/A AB_8395040 WTE6260
F4/80 unconjugated Cell Signaling Technology 71299S BM8.1 AB_2938669 1
TotalSeqTM-B 0301 Hashtag 1 (CD45 and MHCI) oligo-tagged BioLegend 155831 30F11 and M1/42 AB_2814067 B376605
TotalSeqTM-B 0302 Hashtag 2 (CD45 and MHCI) oligo-tagged BioLegend 155833 30F11 and M1/42 AB_2814068 B368762
TotalSeqTM-B 0303 Hashtag 3 (CD45 and MHCI) oligo-tagged BioLegend 155835 30F11 and M1/42 AB_2814069 B367300

CyTOF antibodies:

Antigen Metal Isotope Supplier Catalog number RRID Clone Lot
CD45 Y89 Fluidigm 3089005B AB_2651152 30-F11 0622022
cPARP La139 BD Biosciences 552597 N/A F21-852 N/A
Ly-6G Pr141 Fluidigm 3141008B AB_2814678 1A8 0132018
CD185 Nd142 Fluidigm 3142008B N/A 614641 1082002
CD115 Nd144 Fluidigm 3144012B AB_2895116 AFS98 0282002
F4/80 Nd146 Fluidigm 3146008B AB_2895117 BM8 1901904
CD11b Nd148 Fluidigm 3148003B AB_2814738 M1/70 1401907
CD19 Sm149 Fluidigm 3149002B AB_2814679 6D5 2012553
p53 Nd150 Biolegend 3150024B N/A DO-7 2881605
CD64 Eu151 Fluidigm 3151012B AB_2814680 X54-5/7.1 3181911
Ki67 Sm152 BD 556003 N/A B56 N/A
Mac-2 Eu153 Fluidigm 3153026B AB_2814900 M3/38 0881634
TER-119 Sm154 Fluidigm 3154005B N/A TER-119 0441913
CD14 Gd156 Fluidigm 3156009B AB_2814681 Sa14-2 0041901
PU.1 Gd157 Cell Signaling Technology 22588F N/A 9G7 N/A
CD184 Tb159 Fluidigm 3159030B N/A L276F12 2681909
MIP1beta Gd160 Fluidigm 3160013B N/A D21-1351 2191532
Ly-6C Dy162 Fluidigm 3162014B AB_2922921 HK1.4 3431915
CX3CR1 Dy164 Fluidigm 3164023B AB_2832247 SA011F11 3431916
CD3e Ho165 Fluidigm 3165020B N/A 145-2C11 3181711
GFP Tm169 Fluidigm 3169009B AB_2814899 SF12.4 N/A
CD169 Er170 Fluidigm 3170018B AB_2885022 3D6.112 2621903
CD44 Yb171 Fluidigm 3171003B AB_2895121 IM7 2009641-14
CD86 Yb172 Fluidigm 3172016B AB_2922923 GL1 3121901
CD117 Yb173 Fluidigm 3173004B AB_2811230 2B8 2631811
I-A/I-E Yb174 Fluidigm 3174003B AB_2922924 M5/114.15.2 2631807
prpS6 Yb175 BD Biosciences Custom N/A N7-548 N/A
pCREB Yb176 Fluidigm 3176005A AB_2934290 87G3 0731807
CD11c Bi209 Fluidigm 3209005B AB_2811244 N418 1521804

Validation

Antibodies used for CyTOF:
Commercially available metal-conjugated primary antibodies were used at the manufacturer's suggested concentration, as indicated in Supplementary Table 2. In-house metal-conjugated antibodies were validated using human cells (cell lines or primary cells) known to be positive or negative controls for a given antibody target. Each antibody was titrated in a concentration range from 0.5 to 8 µg/mL, and the lowest concentration that discriminated positive from negative without spillover in another channel was chosen.

- Antibodies used for Flow Cytometry.
All antibodies used herein were validated using several types of positive and negative controls. Positive controls included mouse peripheral blood mononuclear cells (PBMC's) and brain single cell suspensions. Negative controls included fluorescence minus one controls, unstained samples, or cells from a species for the which the antibody is not supposed to show cross-reactivity.

Furthermore, all antibodies used here for flow cytometry have been previously reported and are routinely used. All vendors' (Biolegend, BD biosciences, eBiosciences/Invitrogen/ThermoFisher) report taking quality control measures to ensure that all antibodies sold are valid and reproducible. See https://www.biolegend.com/de-de/quality-control, and https://www.thermofisher.com/us/en/home/life-science/antibodies/invitrogen-antibody-validation.html for details on how each manufacturer validates their antibodies.

Antibodies used for Western blot:
The anti-mouse GRN antibody was validated using samples from Grn+/+ (wild type C57BL/6 mice, Jax strain #000664) and Grn-/- mice (Jax Strain #013175). Samples from Grn+/+ and Grn-/- mice (serum, brain, and eye lysates) are included in each Western blot reported in this work and were used as positive and negative controls, respectively (Fig. 7j, Fig.8c, Supp Fig. 17).
The anti-GFP antibody was validated comparing samples from untreated wild type C57BL/6 mice (Jax strain #000664), untreated Grn-/- mice (Jax Strain #013175), and Grn-/- mice (Jax Strain #013175) transplanted with bone marrow from C57BL/6-Tg(CAG-EGFP) 131Osb/LeySopJ mice (Jax strain #006567). Samples from untreated mice are included in each Western blot reported in this work and

were used as negative controls (Fig.8c, Supp Fig. 17).

Antibodies used for histology:
Negative control slides from each tissue sample stained with secondary antibodies only (no primary antibodies) were processed in parallel with fully stained slides (stained with primary and secondary antibodies) to subtract nonspecific fluorescence signals.

Antigen Conjugation Validation/Reference
BD Fc BloBlock™ unconjugated https://www.bdbiosciences.com/en-eu/products/reagents/flow-cytometry-reagents/research-reagents/single-color-antibodies-ruo/purified-rat-anti-mouse-cd16-cd32-mouse-bd-fc-block.553142
CD45 PE-Cy7 https://www.thermofisher.com/antibody/product/CD45-2-Antibody-clone-104-Monoclonal/25-0454-82
CD45 PE-Cy7
CD11b PE https://www.biolegend.com/en-ie/products/pe-anti-mouse-human-cd11b-antibody-349
TER-119 PE-Cy5 https://www.thermofisher.com/antibody/product/TER-119-Antibody-clone-TER-119-Monoclonal/15-5921-82
Ly6C BV605 https://www.bdbiosciences.com/en-us/products/reagents/flow-cytometry-reagents/research-reagents/single-color-antibodies-ruo/bv605-rat-anti-mouse-ly-6c.563011
CD3 APC https://www.thermofisher.com/antibody/product/CD3-Antibody-clone-17A2-Monoclonal/17-0032-82
CD19 PB https://www.biolegend.com/en-ie/products/pacific-blue-anti-mouse-cd19-antibody-2987
Ly6G PE https://www.biolegend.com/de-de/products/pe-anti-mouse-ly-6g-antibody-4777
CD41 PB https://www.biolegend.com/de-de/products/pacific-blue-anti-mouse-cd41-antibody-16505
CSF1R APC "https://www.thermofisher.com/antibody/product/CD115-c-fms-Antibody-clone-AFS98-Monoclonal/17-1152-82
"
SCA1/Ly-6A/E PE https://www.thermofisher.com/antibody/product/Ly-6A-E-Sca-1-Antibody-clone-D7-Monoclonal/12-5981-81
KIT/CD117 APC https://www.thermofisher.com/antibody/product/CD117-c-Kit-Antibody-clone-2B8-Monoclonal/17-1171-81
CD3 PB https://www.biolegend.com/de-de/products/pacific-blue-anti-mouse-cd3-antibody-3317
TER-119 PB https://www.biolegend.com/de-de/products/pacific-blue-anti-mouse-ter-119-erythroid-cells-antibody-6137
Ly6C PB https://www.biolegend.com/de-de/products/pacific-blue-anti-mouse-ly-6c-antibody-6024
B220 PB https://www.biolegend.com/de-de/products/pacific-blue-anti-mouse-human-cd45r-b220-antibody-2857
Progranulin unconjugated Fig. 7j, Fig.8c, Supp Fig. 17.
GFP unconjugated https://www.thermofisher.com/antibody/product/GFP-Antibody-Polyclonal/A-11122
Alpha tubulin unconjugated https://www.sigmaaldrich.com/US/en/product/sigma/t6199
Cathepsin D unconjugated https://www.rndsystems.com/products/mouse-cathepsin-d-antibody_af1029
Ubiquitin unconjugated https://link.springer.com/article/10.1007/s12035-017-0555-x
IBA1 unconjugated https://labchem-wako.fujifilm.com/us/product/detail/W01W0101-1974.html
F4/80 unconjugated https://www.cellsignal.com/products/primary-antibodies/f4-80-bm8-1-rat-mab/71299
TotalSeqTM-B 0301 Hashtag 1 (CD45 and MHCI) oligo-tagged https://www.biolegend.com/fr-ch/products/totalseq-b0301-anti-mouse-hashtag-1-antibody-17771
TotalSeqTM-B 0302 Hashtag 2 (CD45 and MHCI) oligo-tagged https://www.biolegend.com/en-ie/products/totalseq-b0302-anti-mouse-hashtag-2-antibody-17772
TotalSeqTM-B 0303 Hashtag 3 (CD45 and MHCI) oligo-tagged https://www.biolegend.com/en-ie/products/totalseq-b0303-anti-mouse-hashtag-3-antibody-17773

Animals and other research organisms

Policy information about studies involving animals; ARRIVE guidelines recommended for reporting animal research, and Sex and Gender in Research

Laboratory animals

Species: Mus musculus
Strains:
- adult, 7-12 week-old, C57BL/6J mice (Jax strain #000664)
- adult, 7-12 week-old, homozygous C57BL/6-Tg(CAG-EGFP)131Osb/LeySopJ mice (Jax strain #006567)
- adult, >12 week-old, hemyzygous B6.Cg-Tg(CAG-mRFP1 mice (Jax strain #005884)
- adult, >12 week-old, heterozygous B6.129P2(Cg)-Cx3cr1tm1Litt/J mice (Jax strain #005582)
- adult, 8 week-old, Grn -/- mice: B6(Cg)-Grntm1.1Aidi/J mice (Jax Strain #013175)

Wild animals

This study did not involve wild animals.

Reporting on sex

Experiments included male and female mice based on availability from the vendor or colony. Data from both sexes were reported together, and no analyses were performed based on sex, as we did not observe any sex-dependent impact on microglia replacement following conditioning with busulfan plus PLX3397 (Fig. 1e; the Source Data file includes sex-disaggregated data).
In experiments where the number of C57BL/6 mice is ≥6 we randomly generated cohorts to include about 50% male and 50% female mice per cohort, as reported in the Methods section and Source Data file.
In experiments using Grn -/- mice, the individual mice were divided in three cohorts (sham, untreated, treated), each cohort was randomly generated to contain about 50% male and 50% female mice per cohort, as reported in the Methods section and Source Data file.

Field-collected samples

This study did not involve samples collected from the field

Ethics oversight	All experiments were performed in accordance with National Institutes of Health institutional guidelines and were approved by the University Administrative Panel on Laboratory Animal Care (IACUC 20565 and 33365).

Note that full information on the approval of the study protocol must also be provided in the manuscript.

Flow Cytometry

Plots

Confirm that:

- [x] The axis labels state the marker and fluorochrome used (e.g. CD4-FITC).
- [x] The axis scales are clearly visible. Include numbers along axes only for bottom left plot of group (a 'group' is an analysis of identical markers).
- [x] All plots are contour plots with outliers or pseudocolor plots.
- [x] A numerical value for number of cells or percentage (with statistics) is provided.

Methodology

Sample preparation	The isolation of cells from mouse tissues (brain, spleen, bone marrow, peripheral blood, peritoneum, liver, heart and lung) and their staining for flow cytometry analyses is described in the Methods section. Collection and staining of primitive mouse haematopoietic stem and progenitor cells (HSPCs) in culture is also reported in the Methods sections.
Instrument	- CytoFLEX (Beckman Coulter)<br>- MA900 Multi-Application Cell Sorter (Sony Biotechnology)
Software	Data collection:<br>1. CytExpert Software for the CytoFLEX platform<br>2. MA900 Software for the MA900 Multi-Application Cell Sorter<br><br>Data analyses:<br>FlowJo 10.8.2 (FlowJo, LLC) was used for analysis of flow cytometry data
Cell population abundance	None of the populations analyzed by flow cytometry were rare.
Gating strategy	Figures exemplifying the gating strategies are provided in the Supplementary Information (Supplementary Figures 2 and 6).

- [x] Tick this box to confirm that a figure exemplifying the gating strategy is provided in the Supplementary Information.
